# Supplementary material for: Slow Sulfide Donor GYY4137 Increased the Sensitivity of Two Breast Cancer Cell Lines to Paclitaxel by Different Mechanisms
Source: Biomolecules. 2024 May 31;14(6):651. doi: 10.3390/biom14060651 (PMC11202087; doi:10.3390/biom14060651)
Supplement: Supplementary file 1 [file biomolecules-14-00651-s001.zip › data western blot_Biomolecules_rev.pdf]

Figure S1D

MDA-MB-231

IP_3R1

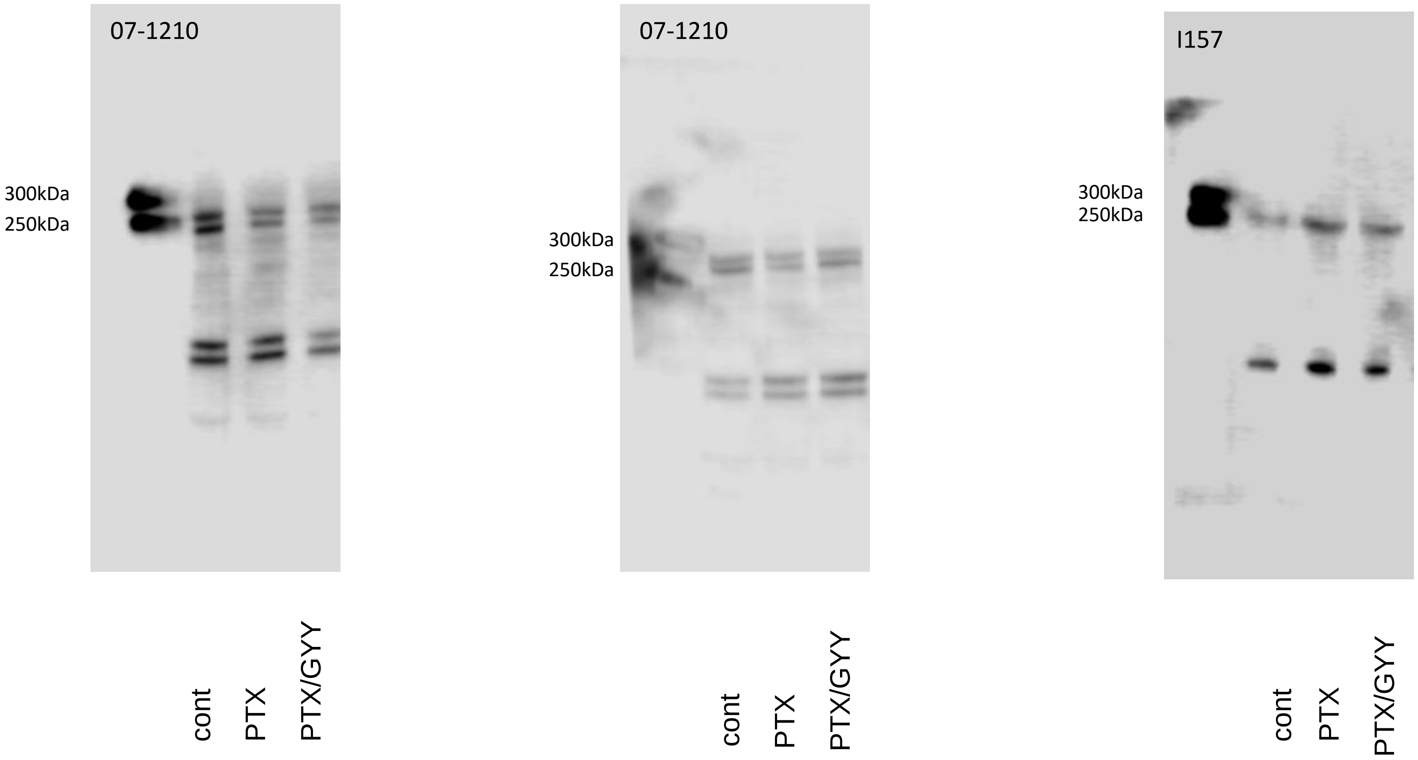

	IP3R1
cont	6390
PTX	5810
PTX/GYY	4550

	IP3R1
cont	771
PTX	676
PTX/GYY	732

	IP3R1
cont	879
PTX	1560
PTX/GYY	1210

Figure S1D

MDA-MB-231

β-actin

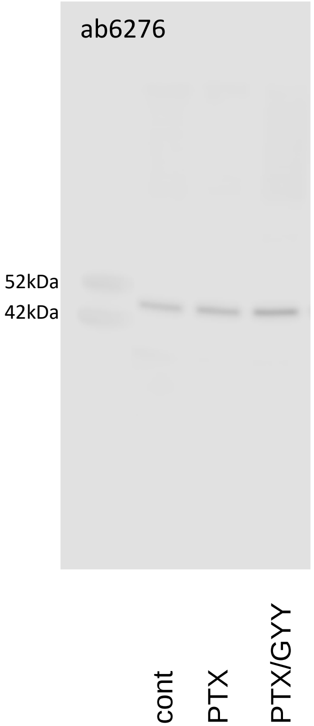

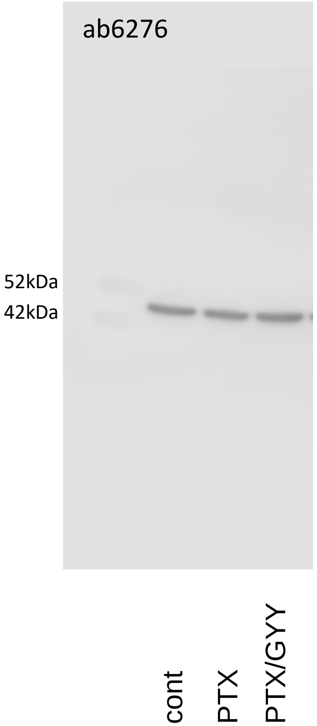

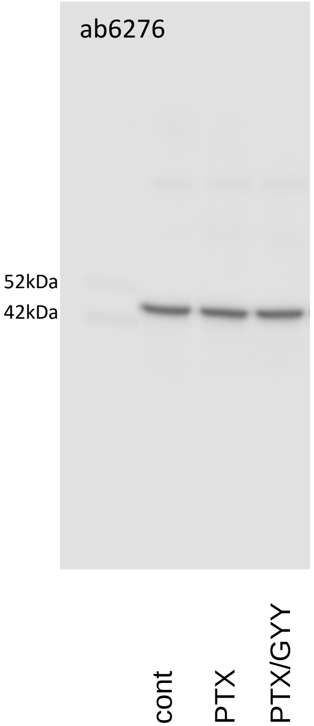

	β-actin
cont	1490
PTX	1950
PTX/GYY	2010

	β-actin
cont	7220
PTX	6940
PTX/GYY	7070

	β-actin
cont	32600
PTX	33100
PTX/GYY	32400

Figure S1D

JIMT1

IP_3R1

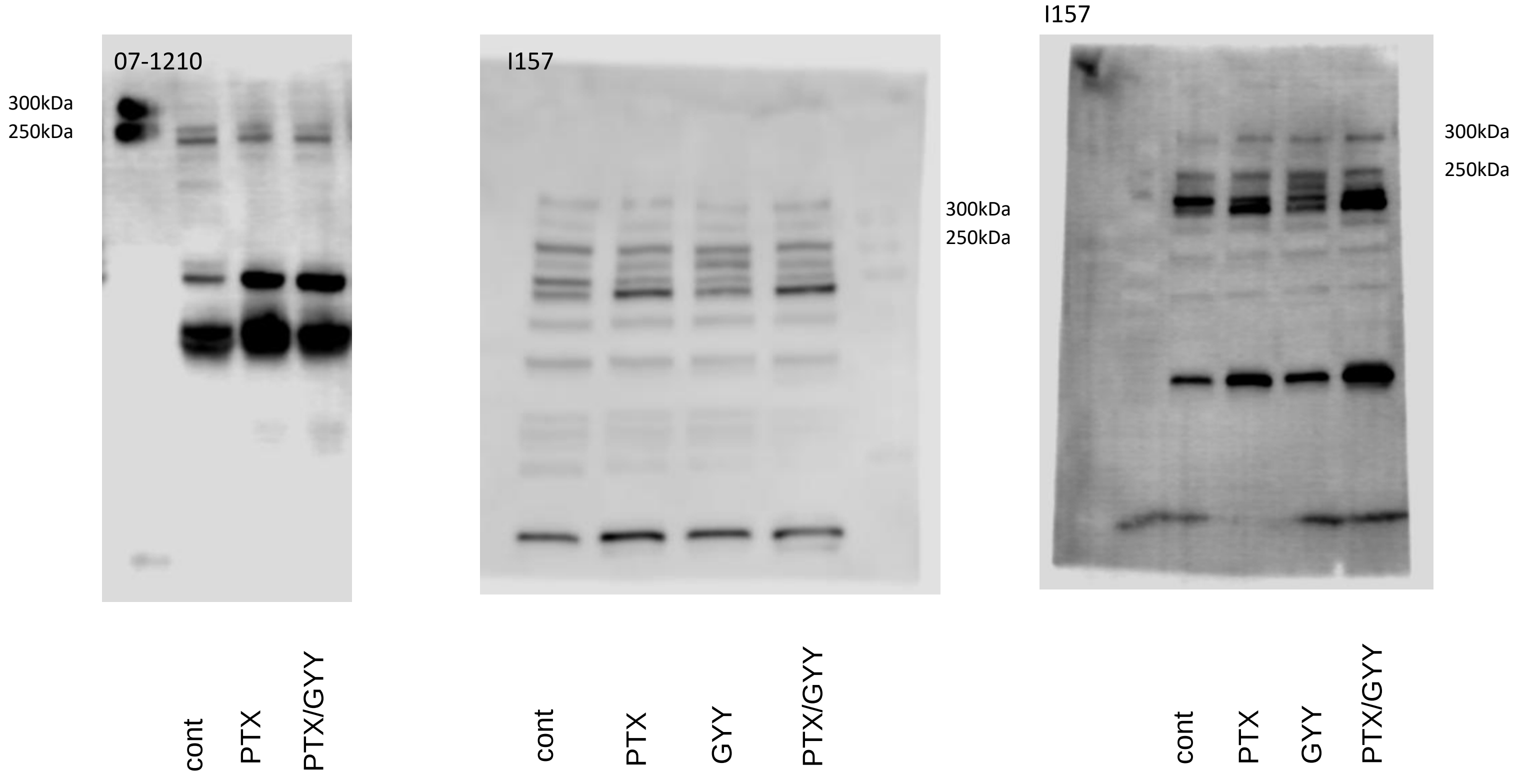

	IP3R1
cont	6210
PTX	5960
PTX/GYY	5620

	IP3R1
cont	690
PTX	640
GYY	670
PTX/GYY	664

	IP3R1
cont	2900
PTX	3400
GYY	3800
PTX/GYY	3500

Figure S1D

β-actin

JIMT1

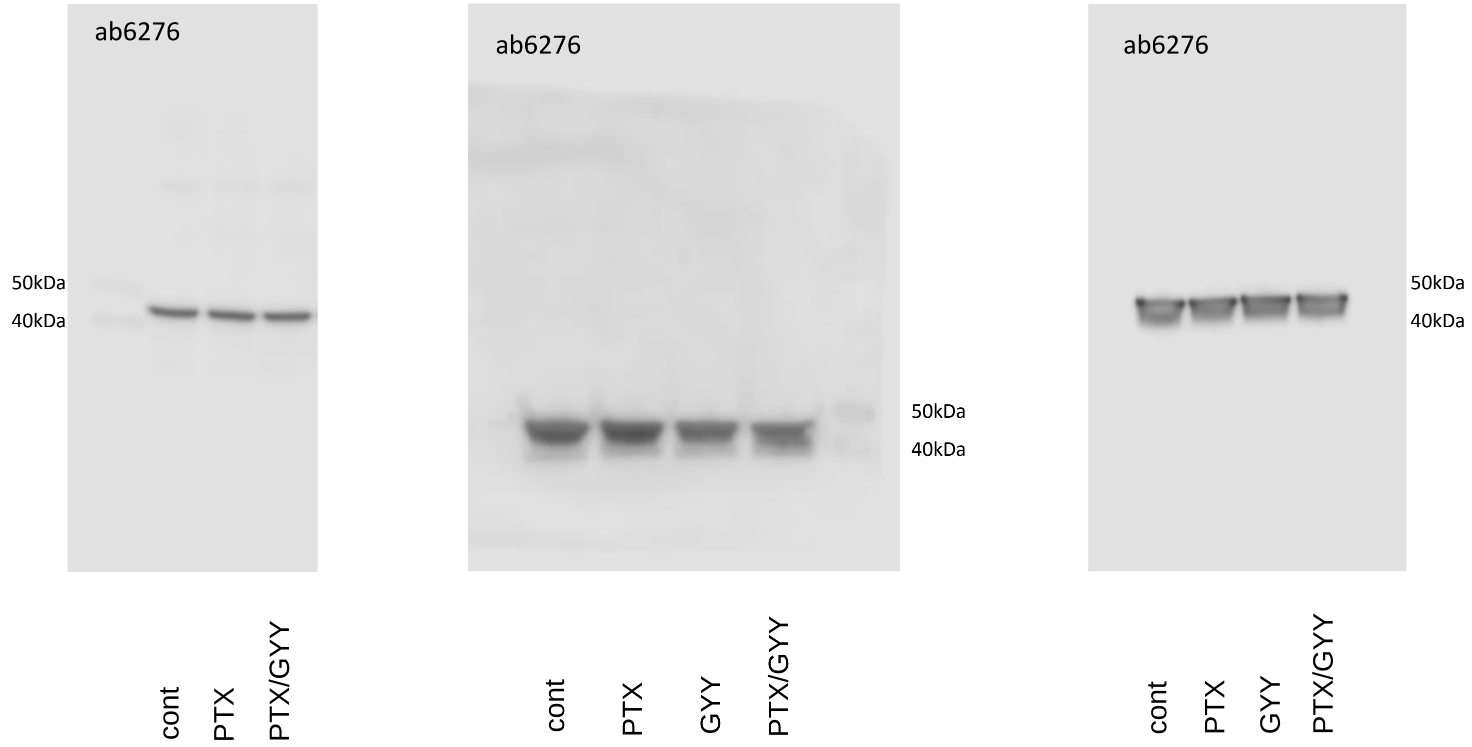

	**β-actin**
cont	326
PTX	331
PTX/GYY	324

	**β-actin**
cont	1400
PTX	1300
GYY	1300
PTX/GYY	1200

	**β-actin**
cont	7300
PTX	6900
GYY	7300
PTX/GYY	7100

Figure S3E

β-tubulin

MDA-MB-231

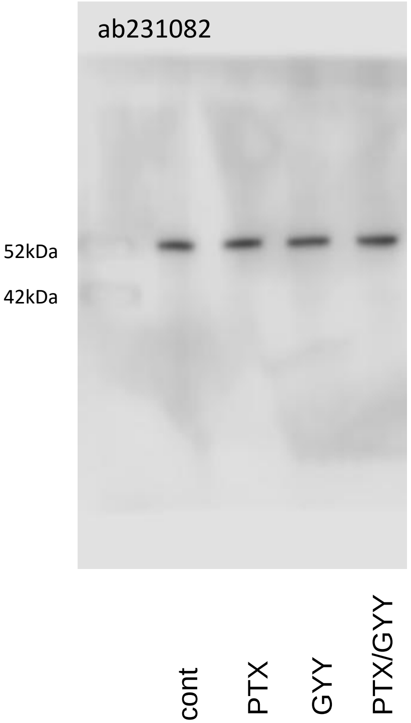

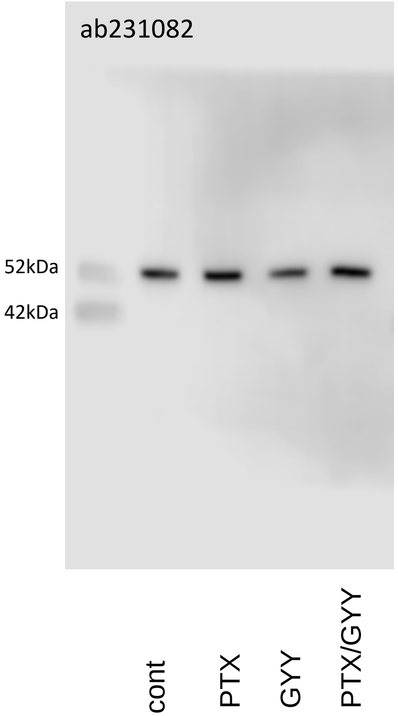

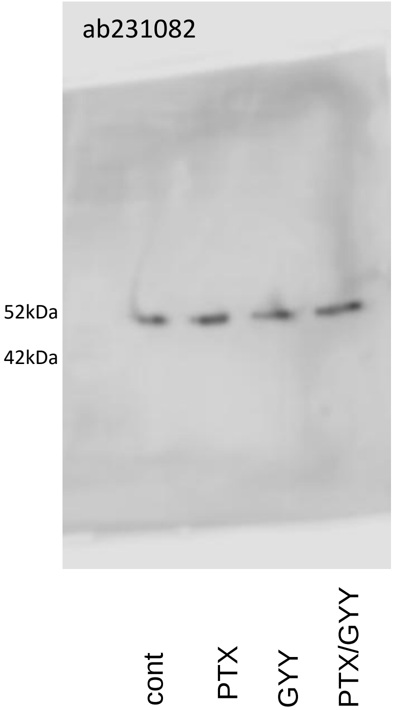

	**β-tubulin**
cont	3050
PTX	3710
GYY	4240
PTX/GYY	4350

	**β-tubulin**
cont	2080
PTX	2710
GYY	1990
PTX/GYY	2830

	**β-tubulin**
cont	2290
PTX	3140
GYY	4000
PTX/GYY	5160

Figure S3E

GAPDH

MDA-MB-231

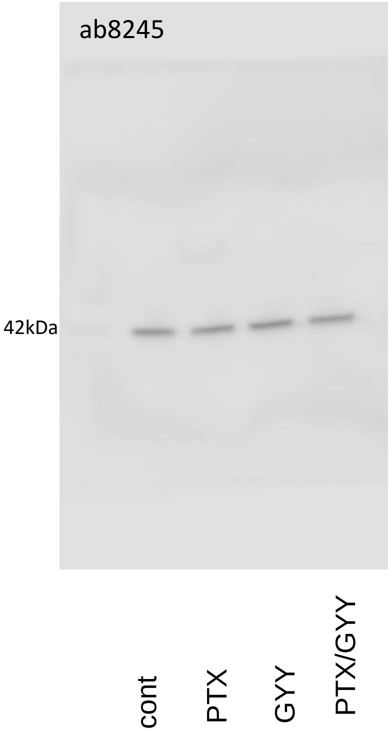

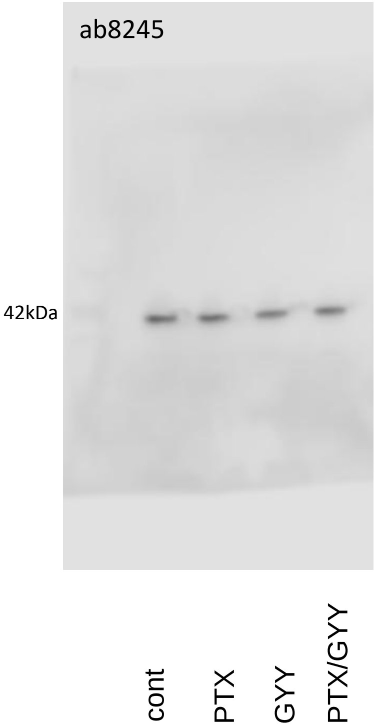

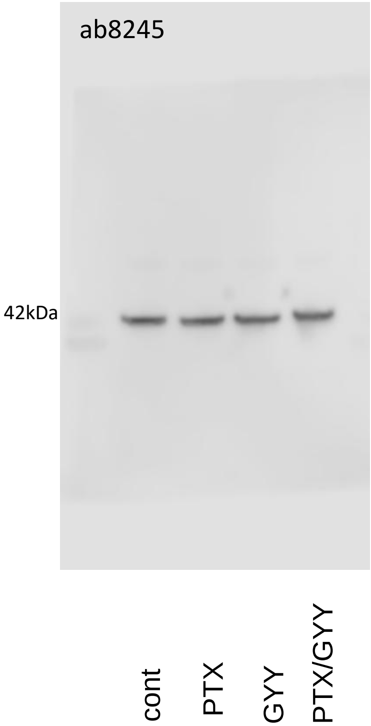

	**GAPDH**
cont	9290
PTX	9470
GYY	10900
PTX/GYY	9970

	**GAPDH**
cont	8250
PTX	7390
GYY	6700
PTX/GYY	6650

	**GAPDH**
cont	8250
PTX	8240
GYY	8300
PTX/GYY	7490

Figure S3E

β-tubulin

JIMT1

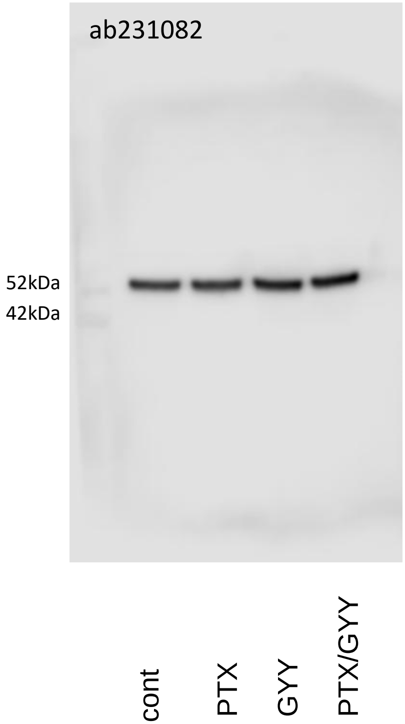

	β-tubulin
cont	78000
PTX	86000
GYY	98000
PTX/GYY	99000

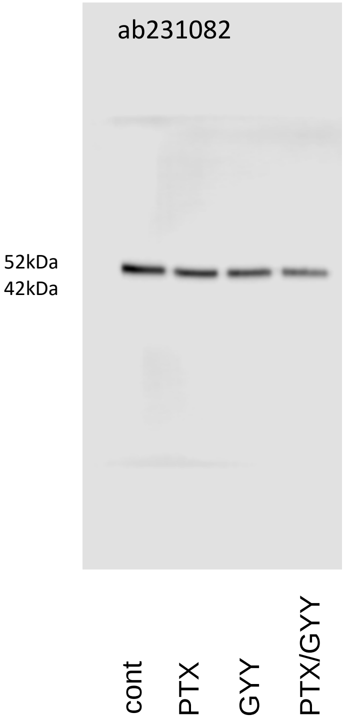

	β-tubulin
cont	32000
PTX	27000
GYY	24000
PTX/GYY	19000

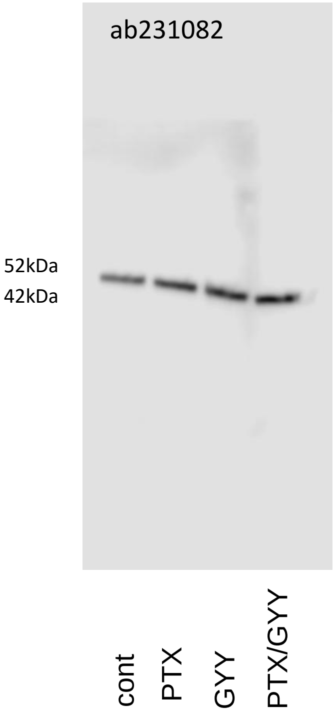

	β-tubulin
cont	21000
PTX	27000
GYY	32000
PTX/GYY	33000

Figure S3E

GAPDH

JIMT1

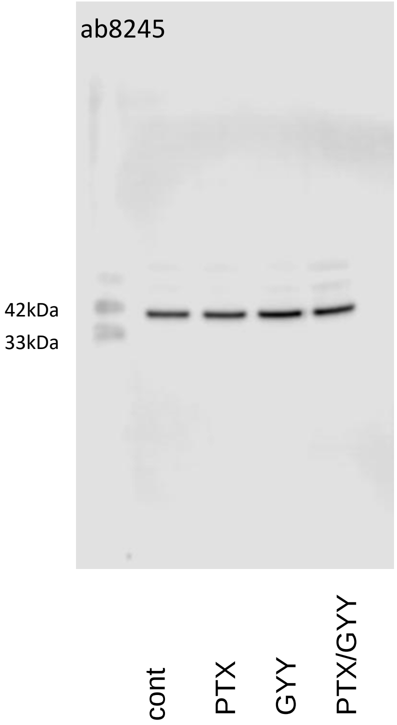

	GAPDH
cont	18000
PTX	19000
GYY	24000
PTX/GYY	20000

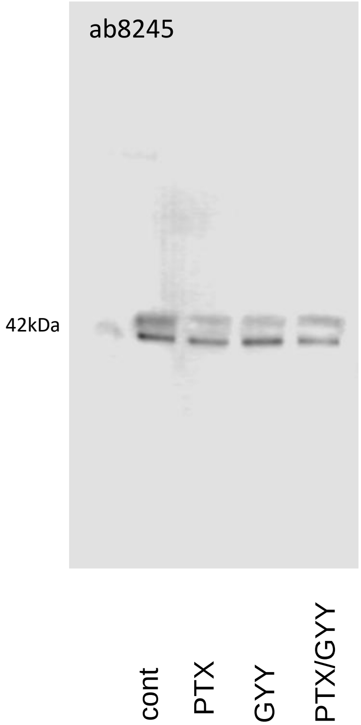

	GAPDH
cont	4000
PTX	3300
GYY	3700
PTX/GYY	2700

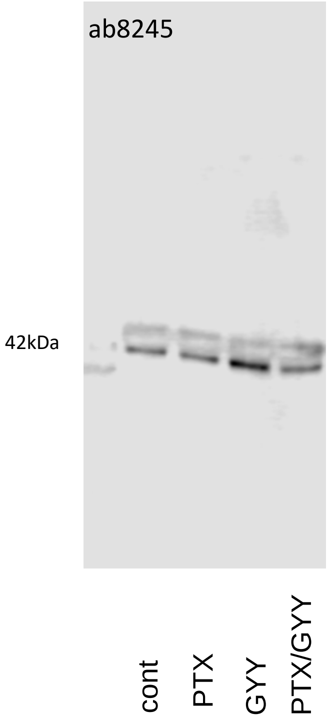

	GAPDH
cont	3500
PTX	3800
GYY	5900
PTX/GYY	4100
