# Supplementary material for: Slow Sulfide Donor GYY4137 Increased the Sensitivity of Two Breast Cancer Cell Lines to Paclitaxel by Different Mechanisms
Source: Biomolecules. 2024 May 31;14(6):651. doi: 10.3390/biom14060651 (PMC11202087; doi:10.3390/biom14060651)
Supplement: Supplementary file 1 [file biomolecules-14-00651-s001.zip › Supplement_Biomolecules_rev_final.pdf]

FigureS1B,C_annexin_MDA-MB-231

cont	PTX2	PTX20	PTX200	GYY100	PTX2+GYY	PTX20+GYY	PTX200+GYY	PTX+DTT	PTX+TCEP
11,03	19,71	26,04	52,35	9,13	22,11	22,5	48,2		
12,32	17,07	20,62	26,64	12,74	23,95	27,69	40,18		
10,35	19,75	22,45	29,35	9,67	24,35	31,87	39,97		
8,11	15,18	26,76	28,98	12,42	21,66	29,72	30,42		
10,26	20,61	32,68	36,41	20,23	21,04	30,17	37,5		
10,25	18,32	9,64	33,28	20,01	27,39	25,32	49,74		
10,02	8,32	9,76	15,54	10,45	33,87	32,34	54,23		
11,97	8,43	14,54	14,95	28,24	28,82	40,88	55,52		
10,01	9,78	21,55	20,46	22,6	31,64	34,12	50,81		
12,5	12,76	18,5	28,72	34,03		30,01	52,89		
8,22	14,64	17,68	39,21	41,7		30,78	58,5		
10,42	12,65	26,07	44,51	40,64		27,08	41,34	24,89	
10,72		28,19	36,54	39,68		26,44	45,05	29,27	
9,66		23,23	36,92	8,22		32,46		21,66	25,89
9,22		14,36	42,46	18,25		34,92		24,36	26,53
10,5		40,61		18,05		24,09		21,1	27,02
10,23		14,55		15,73		25,79		22,54	24,33
10,19		19,89		10,92				23,75	
12,58		17,62		10,68				21,67	
9,16		18,83		9,22		15,58		24,3	24,78
7,9		16,72		10,07		15,22		22,21	22,56
9,25		14,43		11,91		12,9		21,31	
11,63		12,83		9,97				21,64	
10,43		12,72		8,83				25,14	
11,55		12,08		8,8				21,21	
9,66		12,33		10,62				24,54	
10,47		11,9		9,35				21,08	
11,84								21,97	
								22,21	
								23,89	

	cont	PTX2	PTX20	PTX200	GYY100	PTX2+GYY	PTX20+GYY	PTX200+GYY	PTX+DTT	PTX+TCEP
mean	10,37	14,77	19,13	32,42	17,12	26,09	27,49	46,49	23,09	25,19
SD	1,26	4,44	7,40	10,51	10,60	4,60	6,98	8,21	2,05	1,64
S.E.M.	0,24	1,28	1,42	2,71	2,04	1,53	1,56	2,28	0,47	0,67

FigureS1B,C_annexin_JIMT1

cont	PTX2	PTX20	PTX200	GYY100	PTX2+GYY	PTX20+GYY	PTX200+GYY	PTX+DTT	PTX+TCEP
12,64	15,47	38,61	38,6	17	18,28	34,33	34,85		
13,46	12,2	34,48	35,87	13,28	15,34	36,99	32,94		
12,09	12,67	37,23	34,67	12,94	18,08	30,83	35,83		
11,6	14,27	34,31	41,53	13,01	15,25	38,64	38,65		
13,84	14,53	40,83	38,12	12,61	12,29	34,03	38,95		
10,89	11,3	34,08	38,78	14,71	11,66	41,38	38,8		
11,02	10,44	28,7	33,93	14,08	10,98	34,69	33,34		
10,22	10,7	31,85	33,43	12,1		31,1	33,48		
7,68	10,57	32,78	36,15	10,88		30,69	34,69		
9,51	15,16	32,5	21,64	21		23,02	18,61		
8,2		19,38	22,28	15,43		24,16	32,07		
10,73		18,86	22	18,98		19,67	21,98		
8,43		27,83							
13,37		24,9							
13,56		29,6		10,48				28,83	48,68
13,92		30,54		11,21		32,72		28,86	45,32
9,6		35,68		13,42		34,35			
11,93		33,76				27,27		30,08	43,57
9,17		35,8				26,35		32	43,72
9		33,37	25,88			25,36			
7,85		39,53	23,71			31,52		18,1	42,76
11,68		33,48	27,66	11,23		35,41			45,93
10,71		29,32		16,84		38,01	32,16	20,34	30,51
11,15		27,47		11,02		39,42	32,25		39,76
11,92						38,77	30,1		
10,05		31,73		12,49		34,53		19,95	25,57
10,74		28,89		12,63		30,93			40,38
10,5		26,39				28,09			29,47

	cont	PTX2	PTX20	PTX200	GYY100	PTX2+GYY	PTX20+GYY	PTX200+GYY	PTX+DTT	PTX+TCEP
mean	10,91	12,73	31,55	31,62	13,77	14,55	32,09	32,58	25,45	39,61
SD	1,83	1,98	5,33	7,01	2,82	2,99	5,58	5,68	5,74	7,62
S.E.M.	0,34	0,63	1,03	1,81	0,63	1,13	1,12	1,47	2,17	2,30

Viability of MDA-MB-231_IC50

cont			GYY 10 µM			GYY 100 µM			GYY 1000 µM		
100,000	93,317	100,000	86,968	100,000	97,012	87,741	100,000	94,593	68,056	76,891	87,035
100,000	92,242	100,000	82,903	97,516	93,014	86,061	94,593	89,991	72,457	71,650	86,095
100,337	92,443	100,000	85,624	100,000	89,890	86,598	92,846	85,020	65,839	82,198	83,710
100,000	95,433	99,296	84,012	100,000	88,244	83,038	98,557	84,617	76,521	84,281	82,668
100,000	91,872	100,000	87,707	90,058	87,102	77,865	99,464	87,741	80,115	81,560	81,862
96,978	88,715	100,000	93,115	96,810	88,580	84,952	100,000	90,327	79,847	83,743	81,224
100,000	94,358	92,880	91,637	100,000	89,521	63,656	89,252	81,123	82,164	76,017	88,144
92,175	100,000	100,000	100,000	100,000	100,000	85,591	100,000	100,000	86,800	80,586	95,164
92,519	100,000	92,560	85,315	88,229	100,000	90,132	100,000	91,730	80,924	89,990	92,277
100,000	100,000	92,762	82,766	92,115	100,000	85,882	92,540	89,282	70,907	89,929	91,508
99,137	92,115	95,676	91,386	93,592	92,702	81,410	92,782	100,000	77,626	86,793	82,057
100,000	97,720	96,162	92,479	93,875	92,155	90,739	89,221	91,204	76,452	87,885	87,541
100,000	98,084	94,098	92,681	97,801	88,492	89,059	85,417	89,565	77,808	86,165	92,034
99,076	96,223	96,850	93,390	100,000	94,847	90,233	89,747	93,653	88,816	91,366	83,798
93,126	100,000	97,680	95,191	100,000	91,872	92,600	87,865	87,926	81,430	87,035	100,209
100,000	100,000	98,449	100,290	100,000	97,821	100,108	96,283	100,000	80,762	86,347	92,742
82,86	83,39		73,68	68,02		81,62	71,06		62,40	48,66	
93,10	88,96		97,70	114,20		60,35	57,95		54,20	63,25	
100,00	91,58		97,48	94,26		93,18	75,03		66,15	55,20	
100,00	100,00		88,77	95,00		59,03	61,35		47,03	53,13	
100,00	100,00		93,73	89,79		90,70	93,70		65,10	81,10	
95,69	100,00		98,81	93,26		83,64	90,37		85,24	77,51	
100,00	100,00		98,37	89,95		100,00	84,77		69,68	77,32	
100,00	98,17		79,77	93,46		88,99	73,48		56,08	60,77	
98,20	100,00		98,01	85,32		90,26	75,83		70,73	76,24	
100,00	95,22		99,61	100,00		90,84	81,21		69,57	48,27	

	cont			GYY 10 µM			GYY 100 µM			GYY 1000 µM		
	mean	SD	S.E.M.	mean	SD	S.E.M.	mean	SD	S.E.M.	mean	SD	S.E.M.
	96,904	4,09	0,51	93,175	7,04	0,87	87,417	10,19	1,26	77,715	12,13	1,50
divided by 100 %	3,096			6,825			12,583			22,285		
dead	0,031			0,068			0,126			0,223		

Dm: 66789 µM/L

Viability of JIMT1_IC50

											mean	SD	S.E.M.	divided by 100%	dead
cont	97,4	96,3	100,0	95,2	99,9	92,2	91,7	100,0	100,0	94,1					
	100,0	93,4	100,0	90,8	94,0	100,0	97,0	100,0	100,0	100,0					
	88,9	100,0	100,0	90,8	100,0	92,4	100,0	86,3	100,0	100,0					
	92,5	79,3	100,0	100,0	100,0	100,0	100,0	99,9	100,0	100,0					
	100	99	95	100	100	100	95	98	97	93					
	99	100	100	100	98	100	97	96	100	95					
	97	100	97	95	98	99	95	94	96	100					
	97	94	100	98	96	99	100	100	100	100					
	96,68	96,61	100,00	100,00	100,00	98,00	92,97	93,81	83,66	93,01					
	100,00	93,07	100,00	100,00	100,00	97,25	100,00	97,96	93,41	96,20	97,2	3,8	0,4	2,8	0,028
GYY 10 µM	91,8	93,5	100,0	100,0	100,0	100,0	87,5	100,0	100,0	100,0					
	94,2	98,6	100,0	100,0	100,0	93,5	92,7	97,0	100,0	100,0					
	100,0	84,2	100,0	100,0	100,0	92,6	100,0	88,0	100,0	92,8					
	77,4	80,6	97,2	100,0	86,9	85,9	89,2	81,6	90,5	75,9					
	96	100	93	96	100	100	100	100	100	98					
	91	91	96	100	100	100	100	100	100	94					
	105	93	100	92	97	94	100	100	99	100					
	94	100	93	100	87	91	96	97	92	100					
	100,00	100,00	100,00	100,00	100,00	100,00	100,00	100,00	100,00	100,00					
	100,00	100,00	100,00	100,00	98,63	100,00	100,00	100,00	99,32	98,20	96,5	5,5	0,6	3,5	0,035
GYY 100 µM	96,4	96,8	100,0	95,8	97,2	95,8	92,2	92,4	100,0	100,0					
	92,9	100,0	100,0	100,0	100,0	100,0	98,5	96,5	100,0	100,0					
	72,7	82,2	94,7	92,2	82,5	66,6	89,9	85,5	84,4	90,5					
	74,3	89,8	89,1	88,2	96,4	66,5	91,5	86,9	83,4	89,5					
	100	100	100	100	100	98	97	96	93	88					
	95	89	99	97	98	97	99	96	89	81					
	91	88	84	100	89	89	91	89	88	87					
	82	76	82	84	88	84	89	84	83	80					
	100,00	100,00	100,00	100,00	100,00	96,28	100,00	100,00	89,67	100,00					
	82,66	95,43	100,00	100,00	95,23	100,00	100,00	100,00	95,31	100,00	92,5	7,9	0,8	7,5	0,075
GYY 1000 µM	86,3	100,0	100,0	100,0	99,8	80,6	74,5	92,0	100,3	100,0					
	74,6	73,4	100,0	89,1	96,6	74,1	78,2	88,3	97,1	98,7					
	44,2	64,0	72,3	72,7	69,4	60,4	72,7	62,4	64,0	76,2					
	57,6	73,4	82,1	87,6	91,1	68,4	90,2	75,3	83,4	83,5					
	96	94	98	98	100	94	98	95	92	92					
	10	77	69	85	99	77	81	84	88	92					
	91	78	79	72	75	77	80	78	79	77					
	79	80	84	76	85	75	82	92	86	81					
	74,13	70,13	81,55	84,18	72,87	73,55	86,07	91,47	72,27	93,57					
	66,74	67,55	76,14	68,79	68,39	76,04	78,62	85,31	70,41	80,32	81,2	13,5	1,3	18,8	0,188

Dm: 41930 µM/L

Necrosis_MDA-MB-231

cont	PTX 20nM	GYY 100 µM	GYY/PTX	DTT 1mM	DTT/PTX	TCEP 1mM	TCEP/PTX
5,34	14,37	2,8	10,44	2,16	14,73	3,12	3,47
3,69	6,25	4,68	6,29	3,47	15,08	3,3	4,29
	4,38	6,09	9,63	2,39	6,41	4,54	3,95
2,22	9,53	3,79	12,28	2,2	8,91	3,48	3,87
3	4,19	4,82	8,24	7,39	7,55	4,73	4,08
3,22	4,44	3,3	11,78	7,94	6,56	7,72	3,98
2,1	1,41	1,86	0,76	6,02	7,41	2,47	4,55
2,46	2,26	2,43	0,91	6,56	7,19	2,83	4,63
2,83	2,08	2,57	1,24	3,47		3,17	3,4
1,92	0,88	0,43	0,52	6,93		2,27	7,4
4,31	0,62	0,49	0,46	2,92		2,88	7,26
1,55	0,57	0,53	0,46	2,09		3,93	3,26
1,55	1,53	1,63	1,57	5,38		3,91	4,52
2,09	2,29	2,84	1,46	5,97		4,39	4,72
2,14	1,72	2,22	4,3			1,87	9,14
4,03	3,71	4,29	4			2,44	
2,78	2,91	4,08	5,59			3,23	
4,09	4,44	4,52	8,37			2,81	
4,42	2,87	4,83	3,26			3,69	
3,13	2,54	4,02	4,52			2,55	
4,54	4,2	4,27	7,72				
2,04	1,46	4,49	0,8				
1,61	1,01	3,63	1,22				
1,2	1,3	3,82	1,15				
2,41	1,93	3,27	4,69				
3,15	3,74	2,68	3,33				
2,33	2,42	2,09	3,41				
2,9	2,79	2,31	3,75				
3,01	2,64	2,04					
3,16	2,29	2,21					
3,98	2,09	2,35					
4,93	3,3						
4,4	3,75						
9	3,73						
3,88	3,57						
2,83	3,58						
2,82	3,96						
2,32	3,3						
2,46	3,65						
2,34	3,56						
2,17	4,33						
3,3	5,61						
5,13	3,18						
4,29	2,17						
6,06	2,66						
4,29	2,73						
4,14	3,01						
2,54							
2,61							
2,09							
2,53							
3,38							
2,36							

	cont	PTX 20 nM	GYY 100 µM	GYY/PTX	DTT 1mM	DTT/PTX	TCEP 1mM	TCEP/PTX
mean	3,21	3,30	3,08	4,36	4,64	9,23	3,47	4,83
SD	1,35	2,28	1,38	3,67	2,17	3,58	1,27	1,71
S.E.M.	0,19	0,33	0,25	0,69	0,58	1,27	0,28	0,44

Necrosis_JIMT1

cont	PTX 20nM	GYY 100 µM	GYY/PTX	DTT 1mM	DTT/PTX	TCEP 1mM	TCEP/PTX
6,35	4,83	3,34	3,99	4,07	12,68	1,47	3,37
4,91	5,72	3,05	5,67	3,1	8,4	7,88	3,45
3,78	6,67	3,93	4,46	3,21	7,25	1,83	5,69
2,65	4,63	2,42	4,99	3,41	7,9	1,45	12,82
3,52	5,55	2,68	4,68	5,72	8,64	1,1	4,49
3,51	5,34	2,78	4,91	6,27	8,68	1,72	3,25
3,17	4,95	3,14	4,33	9,51	11,98	2,56	7,68
3,73	5,73	3,04	4,51			8,6	14,52
3,65	6,25	3,37	4,93			7,27	7,04
2,9	1,57	1,07	3,02			2,04	17,53
6,77	2,68	1,08	3,3				11,21
2,59	4,19	8,81	2,74				17
1,53	3,38	5,22	0,94				
7,1	4,93	3,03	0,74				
4,2	2,82	1,97	1,25				
7,49	2,97	4,21	17,57				
5,58	7,91	9,59	18,96				
6,74	3,37	6	22,07				
10,06	4,25	6,18	6,18				
1,69	13,59	6,44	6,44				
4,01	15,19	6,23	6,23				
2,82	5,69	4,47	12,27				
6,9	6,37	4,25	10,03				
4,23	7,29	5,32					
3,79	5,49	4,1					
3,24	12,17						
4,38	17,7						
2	13,59						
5,06	16,97						
7,35	7,55						
7,55	8,29						
5,4	5,86						
4,85	4,75						
4,23	4,35						
2,85	5,73						
2,61	4,41						
3,29	6,54						
4,25	11,21						
7,95	7,05						
4,92	6,9						
6,21							
4,79							
5,55							

	kontrola	PTX 20nM	GYY100uM	GYY/PTX	DTT1mM	DTT/PTX	TCEP1mM	TCEP/PTX
mean	4,65	6,86	4,23	6,70	5,04	9,36	3,59	9,00
SD	1,91	3,90	2,29	5,30	2,33	2,10	3,03	5,39
S.E.M.	0,29	0,61	0,46	1,10	0,88	0,79	0,96	1,55

JIMT-1 control

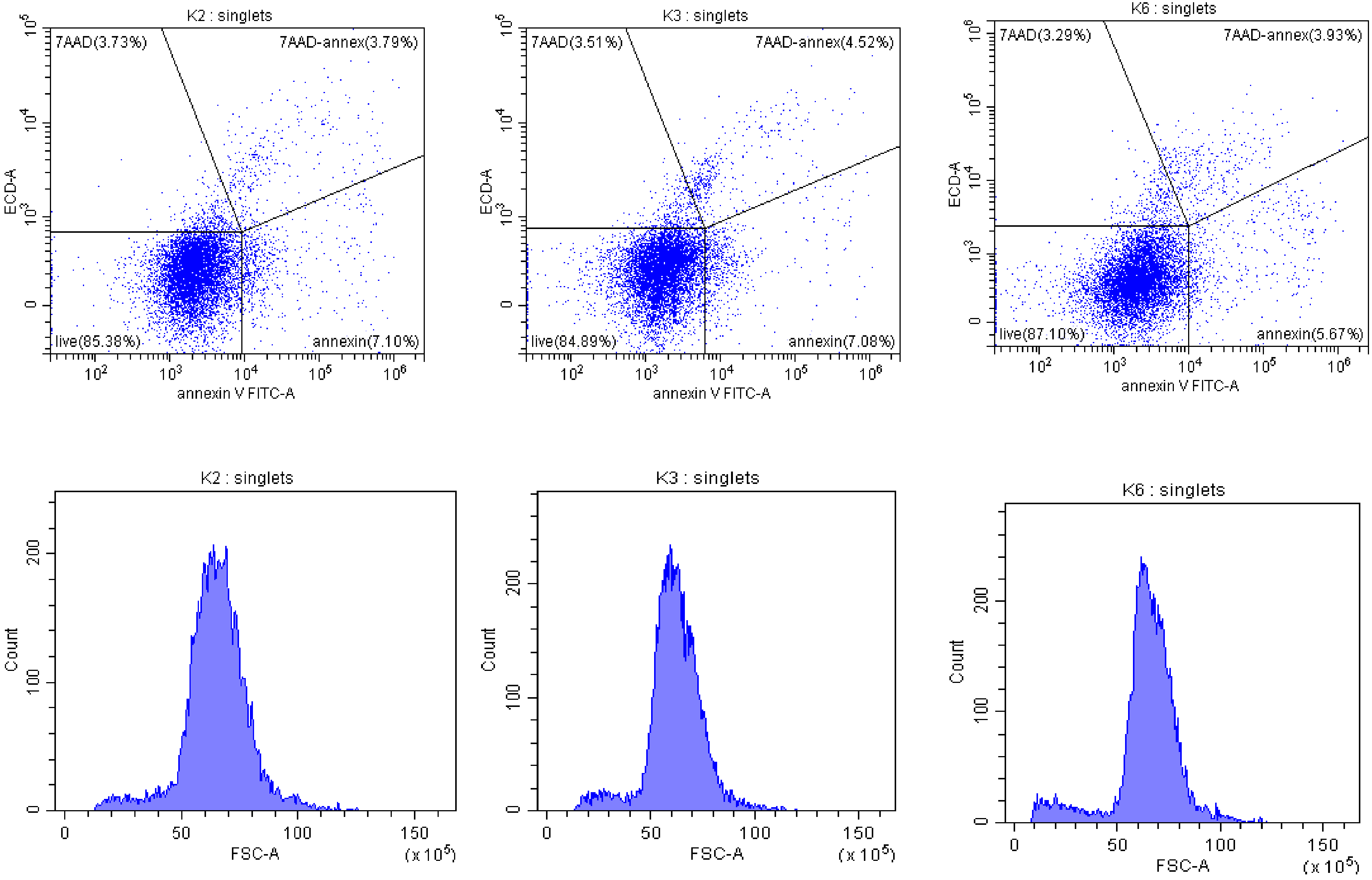

JIMT-1 PTX 2nM

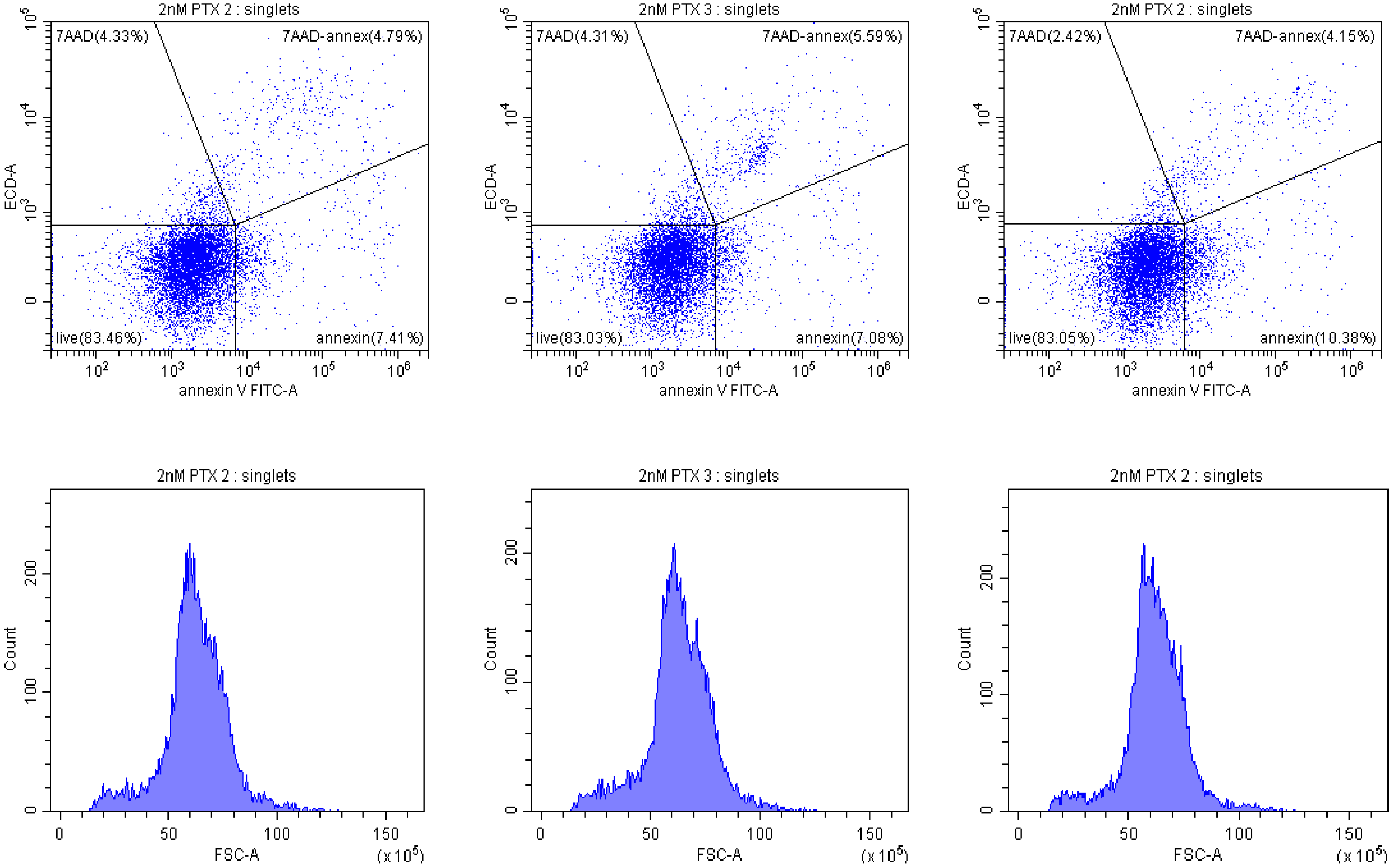

JIMT-1 PTX 20nM

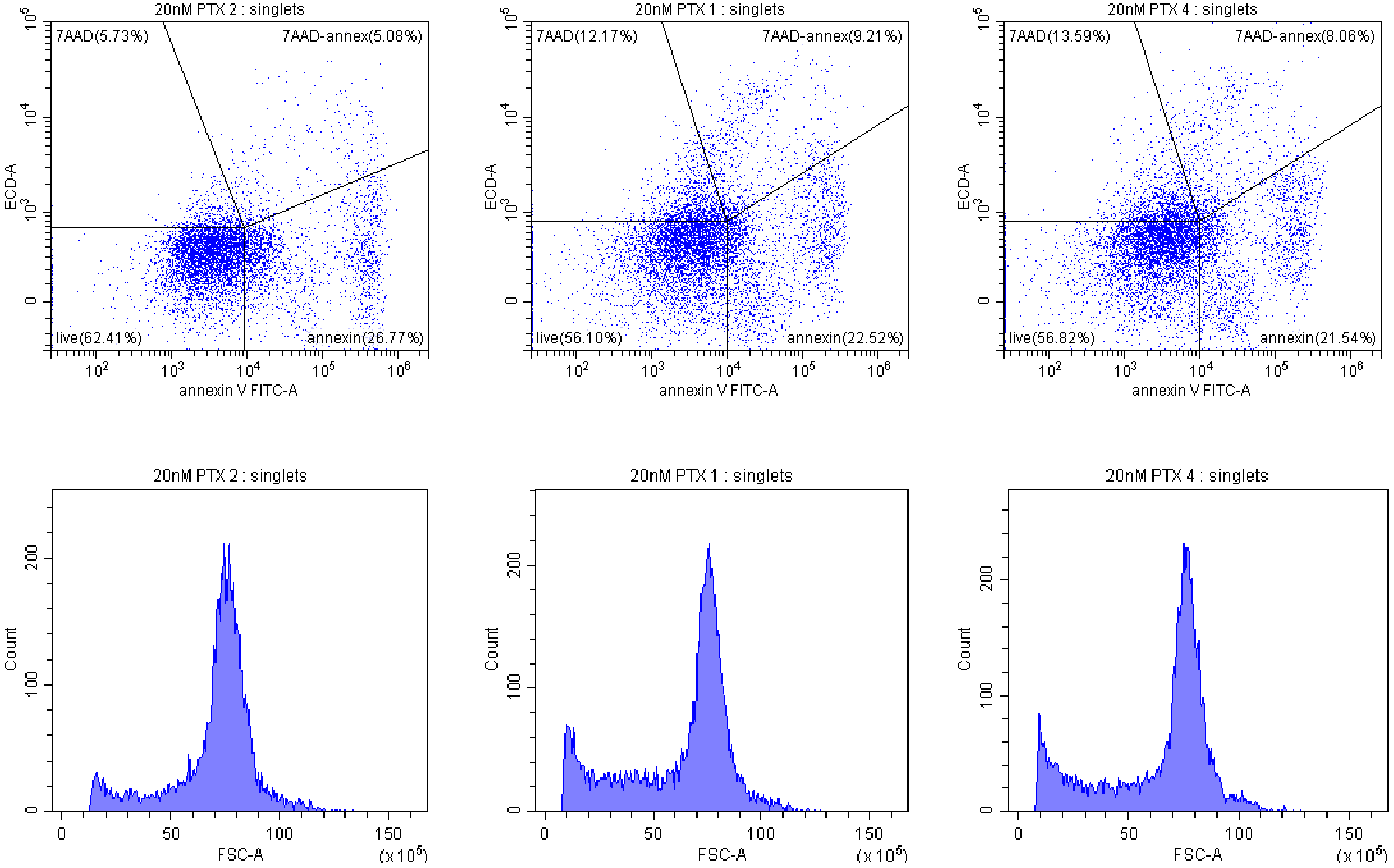

JIMT-1 PTX 200nM

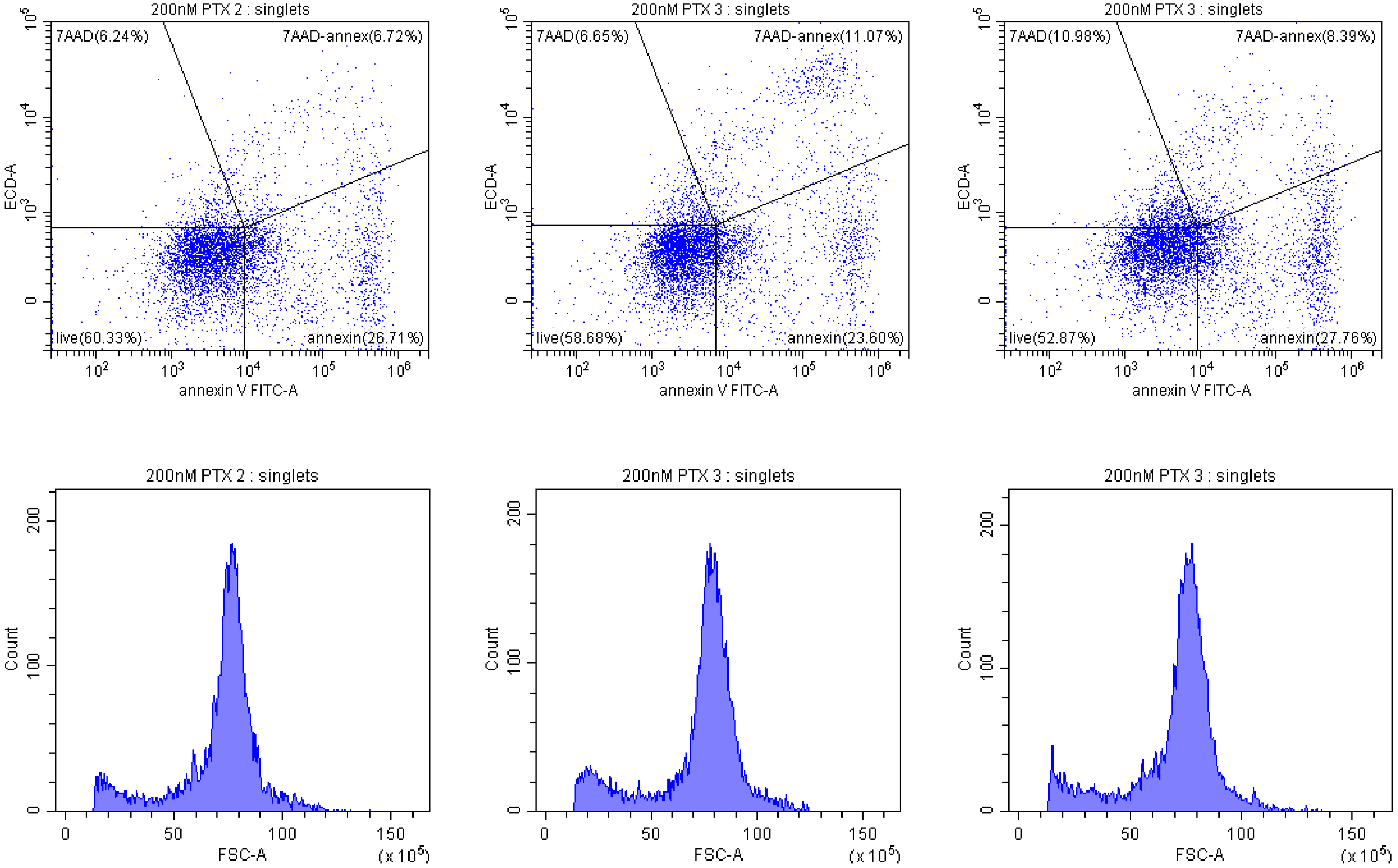

JIMT-1 GYY 100μM

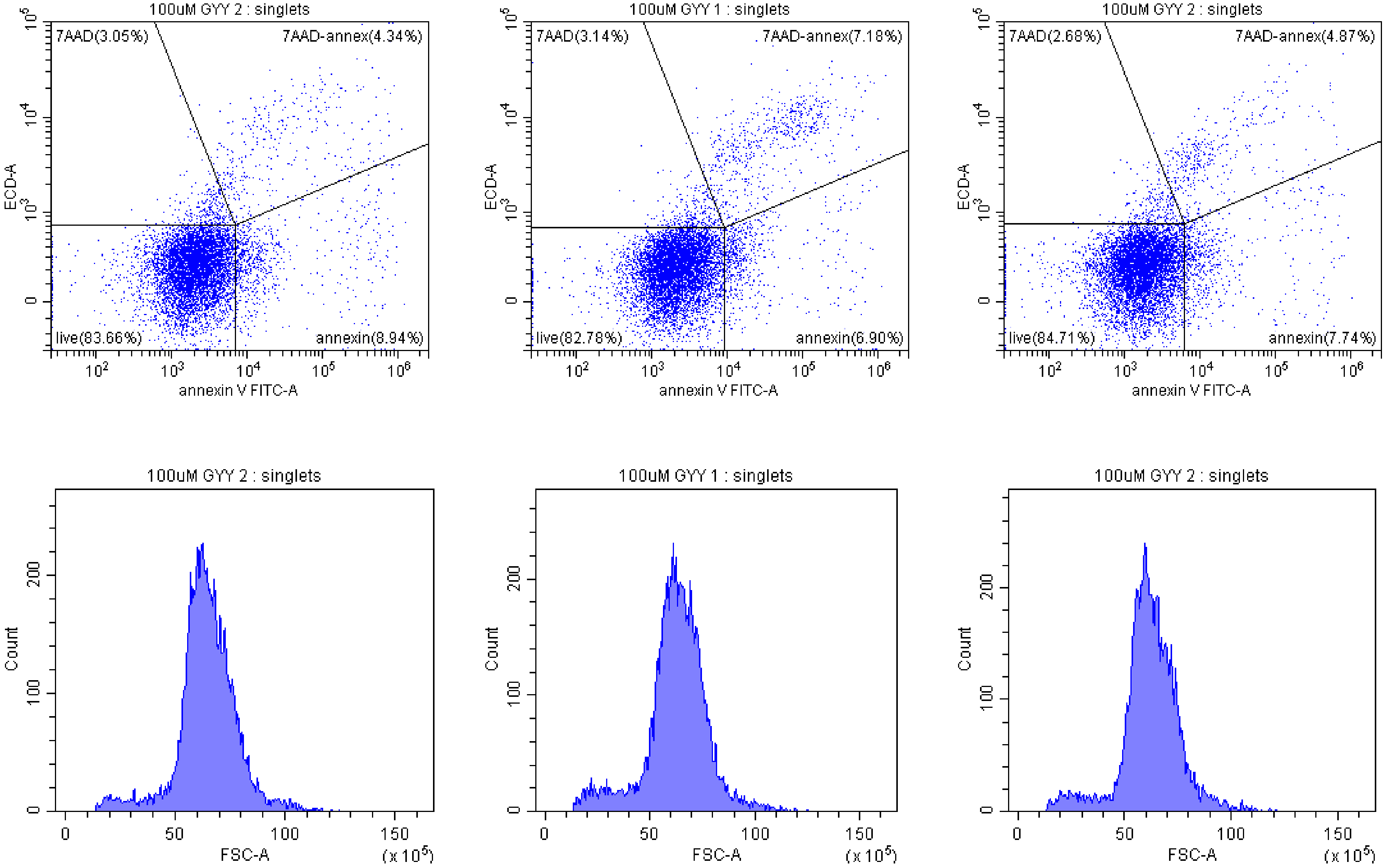

JIMT-1 PTX 2 nM+GYY 100µM

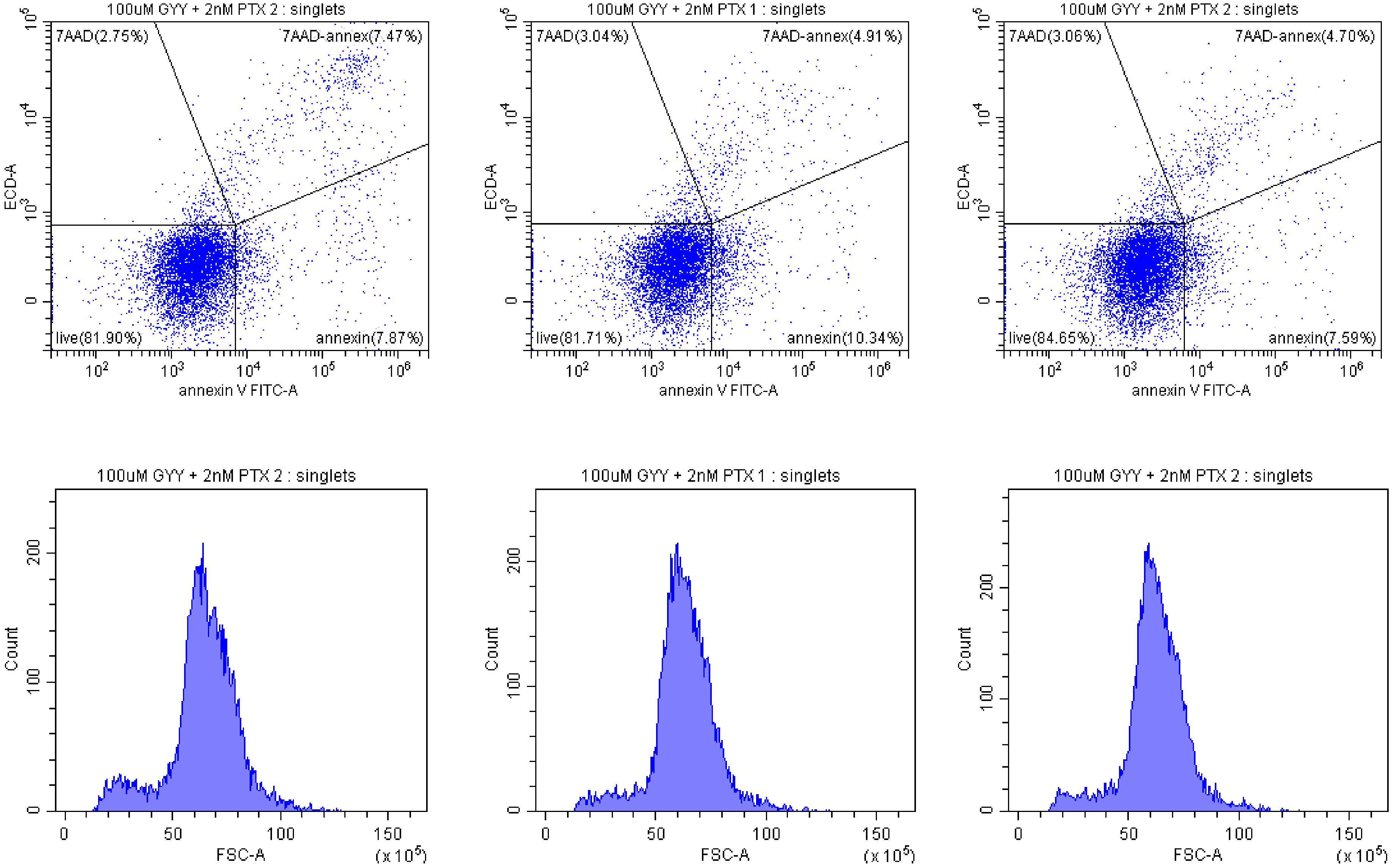

JIMT-1 PTX 20 nM+GYY 100µM

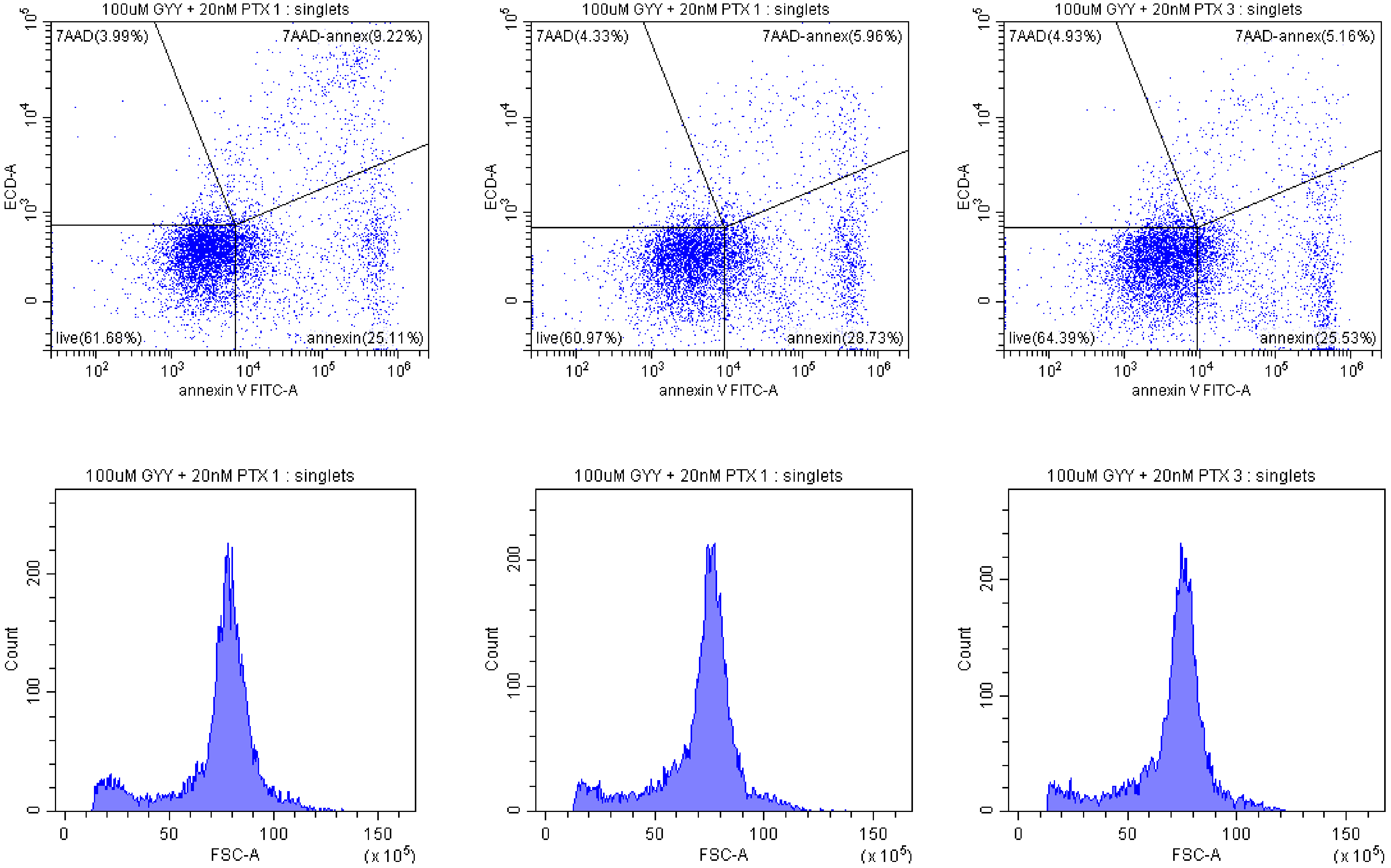

JIMT-1 PTX 200 nM+GYY 100µM

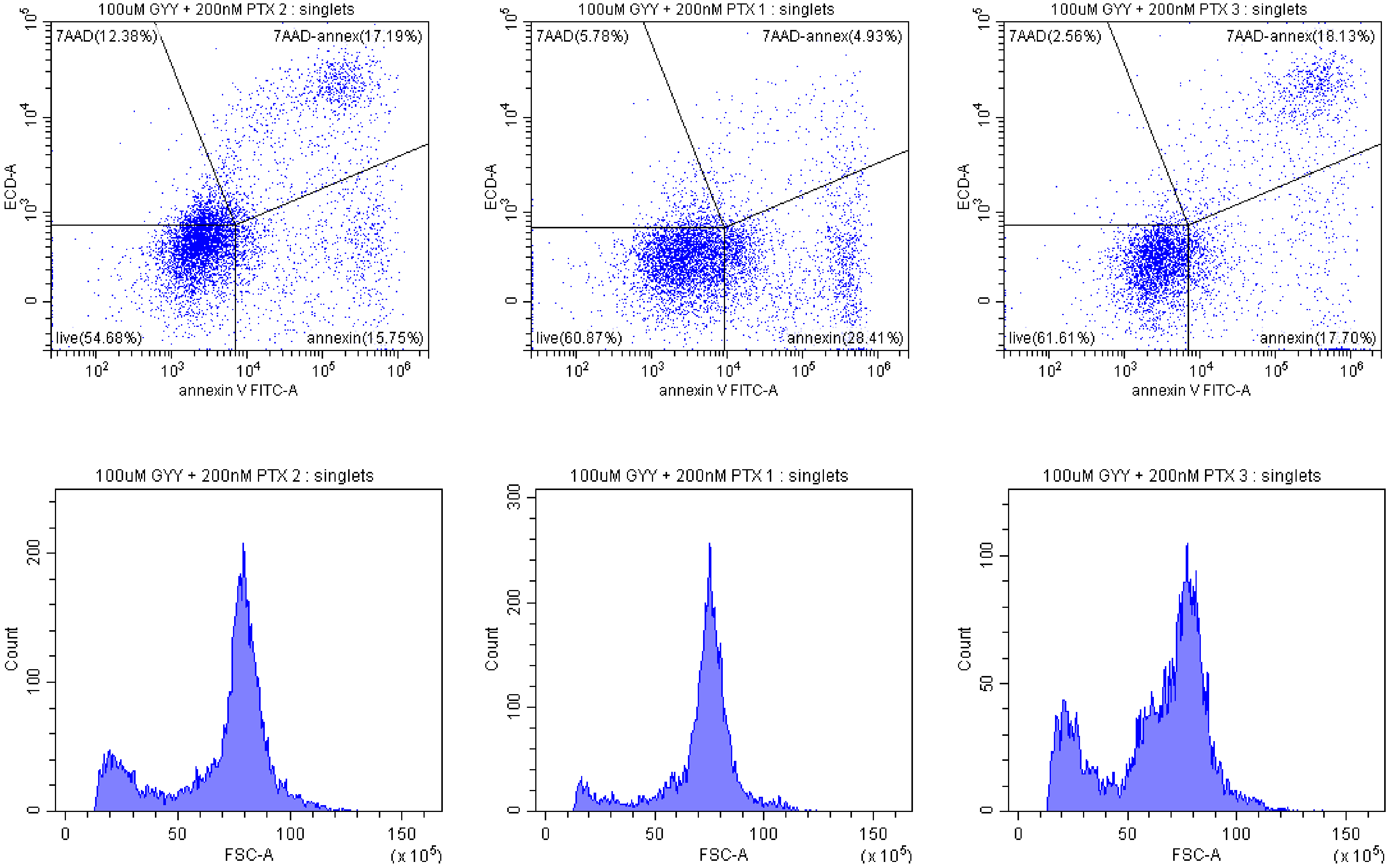

JIMT-1 DTT 1 mM

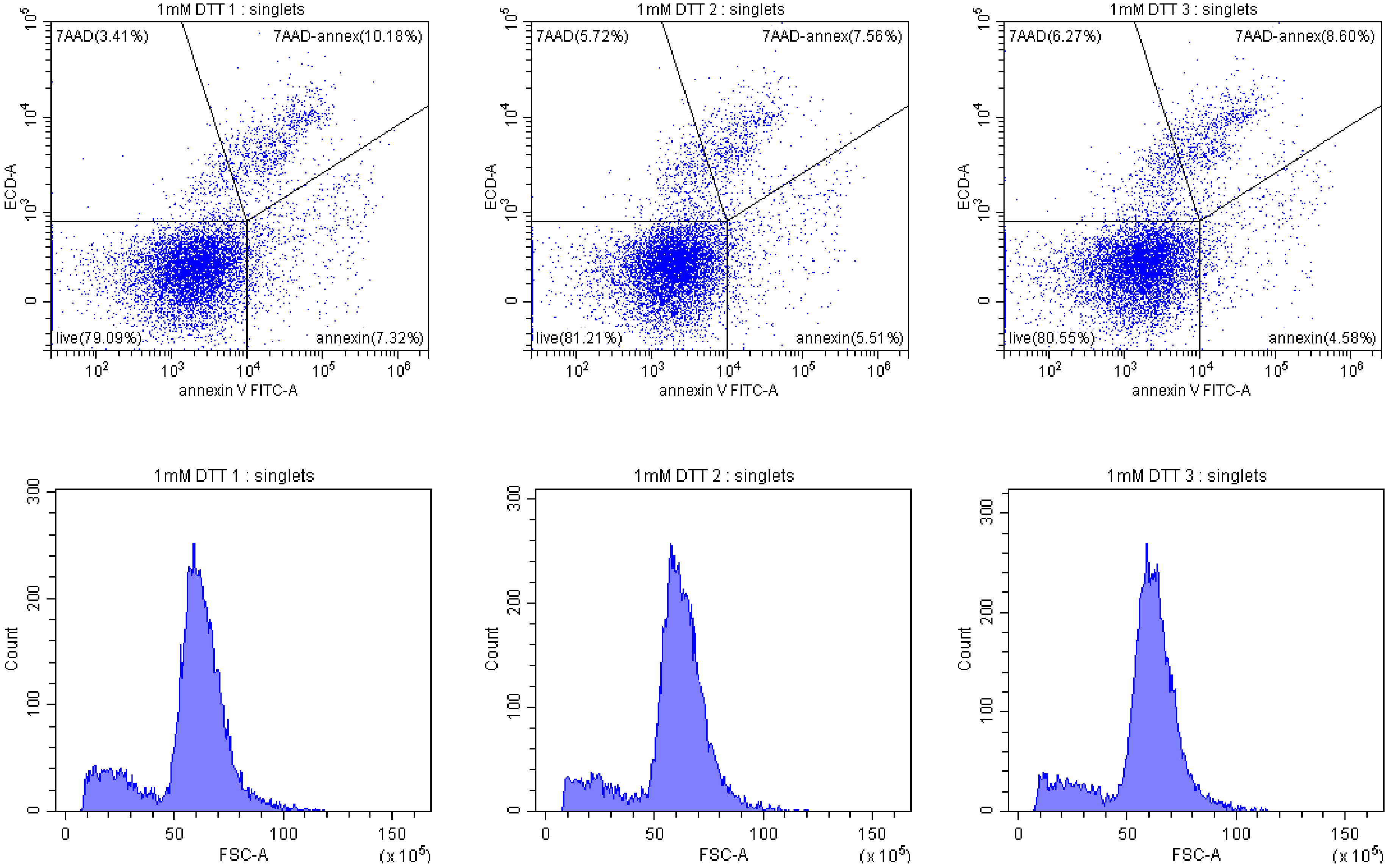

JIMT-1 PTX 20 nM+DTT 1 mM

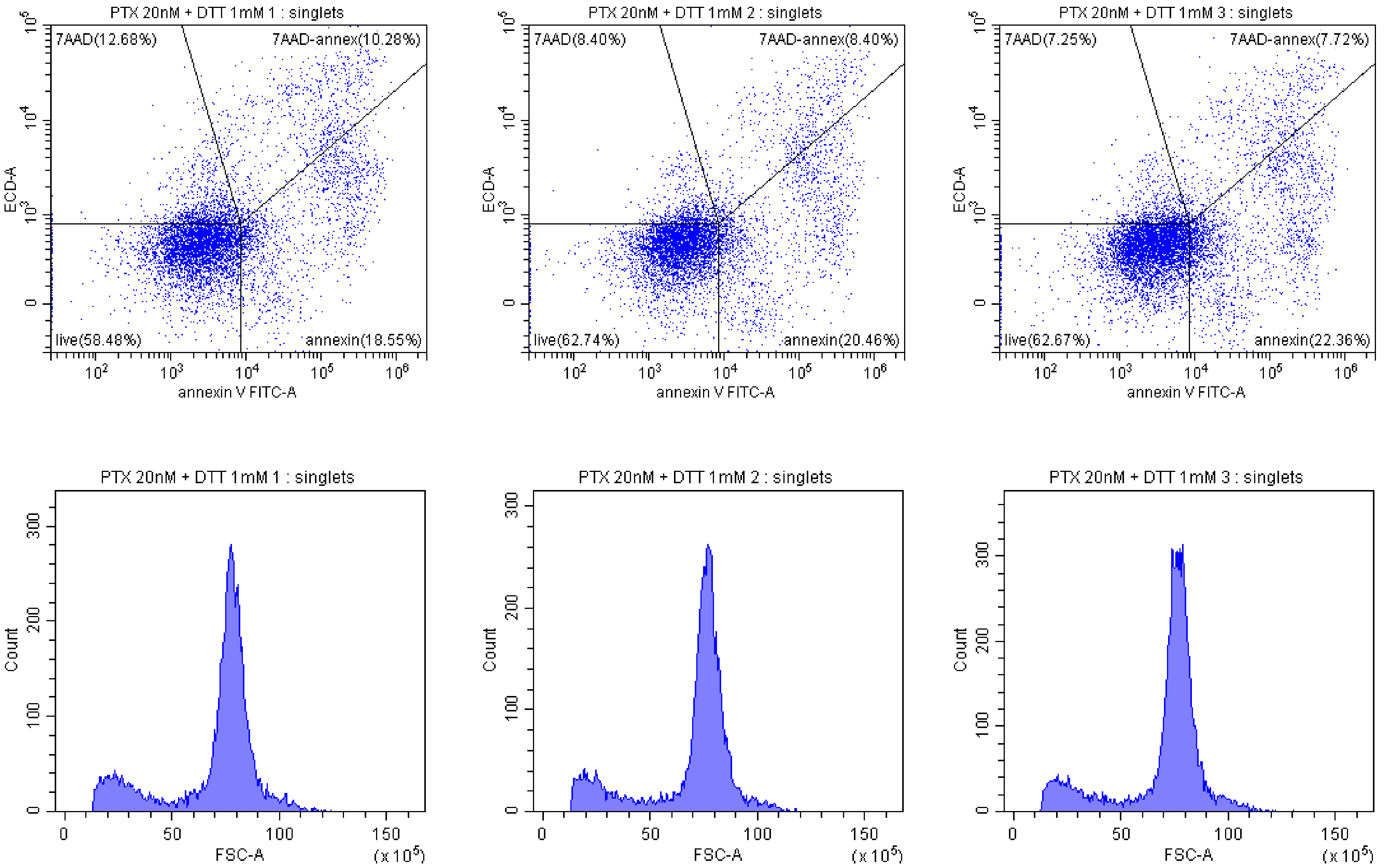

JIMT-1 TCEP 1 mM

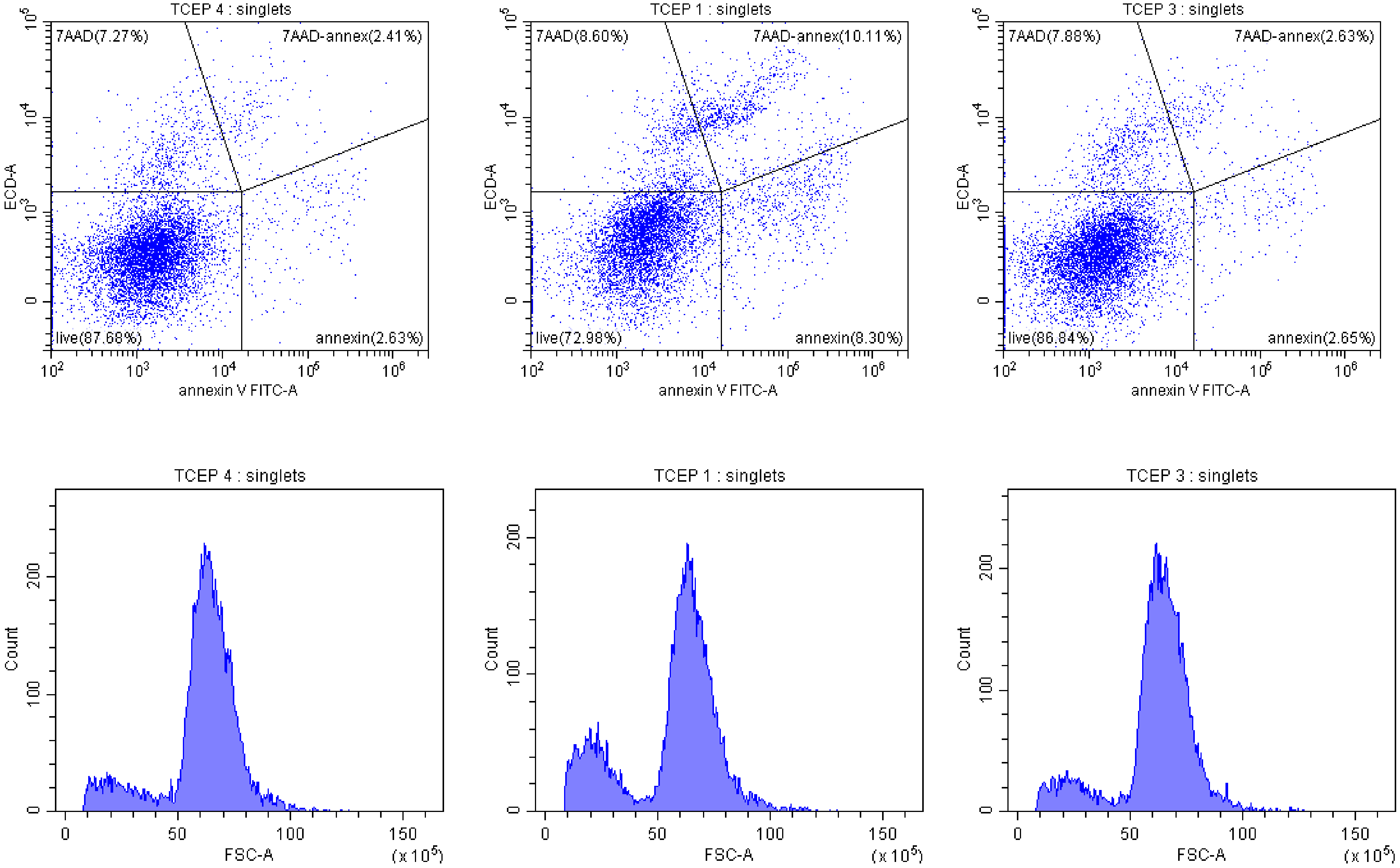

JIMT-1 PTX 20 nM+TCEP 1 mM

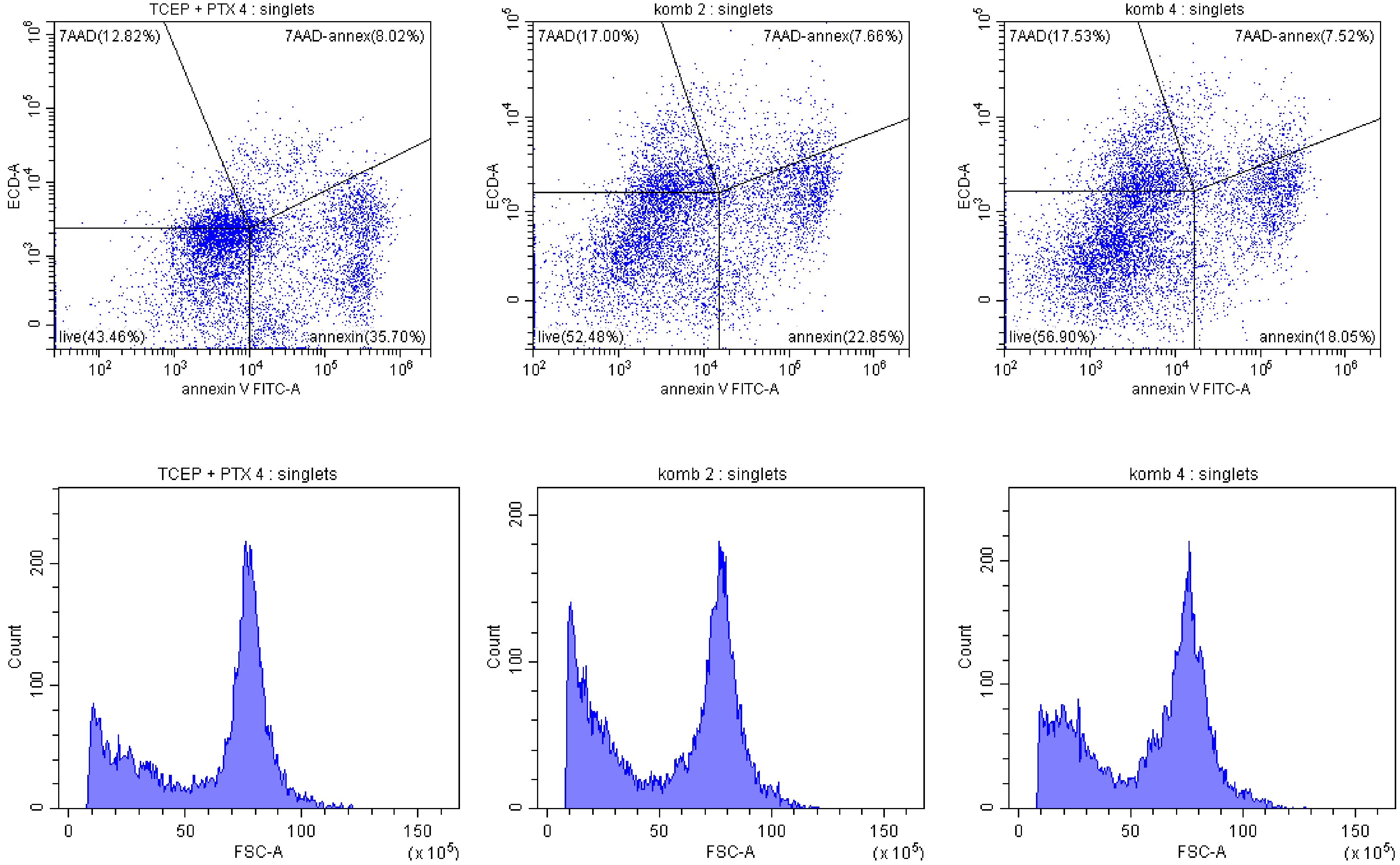

MDA-MB-231 control

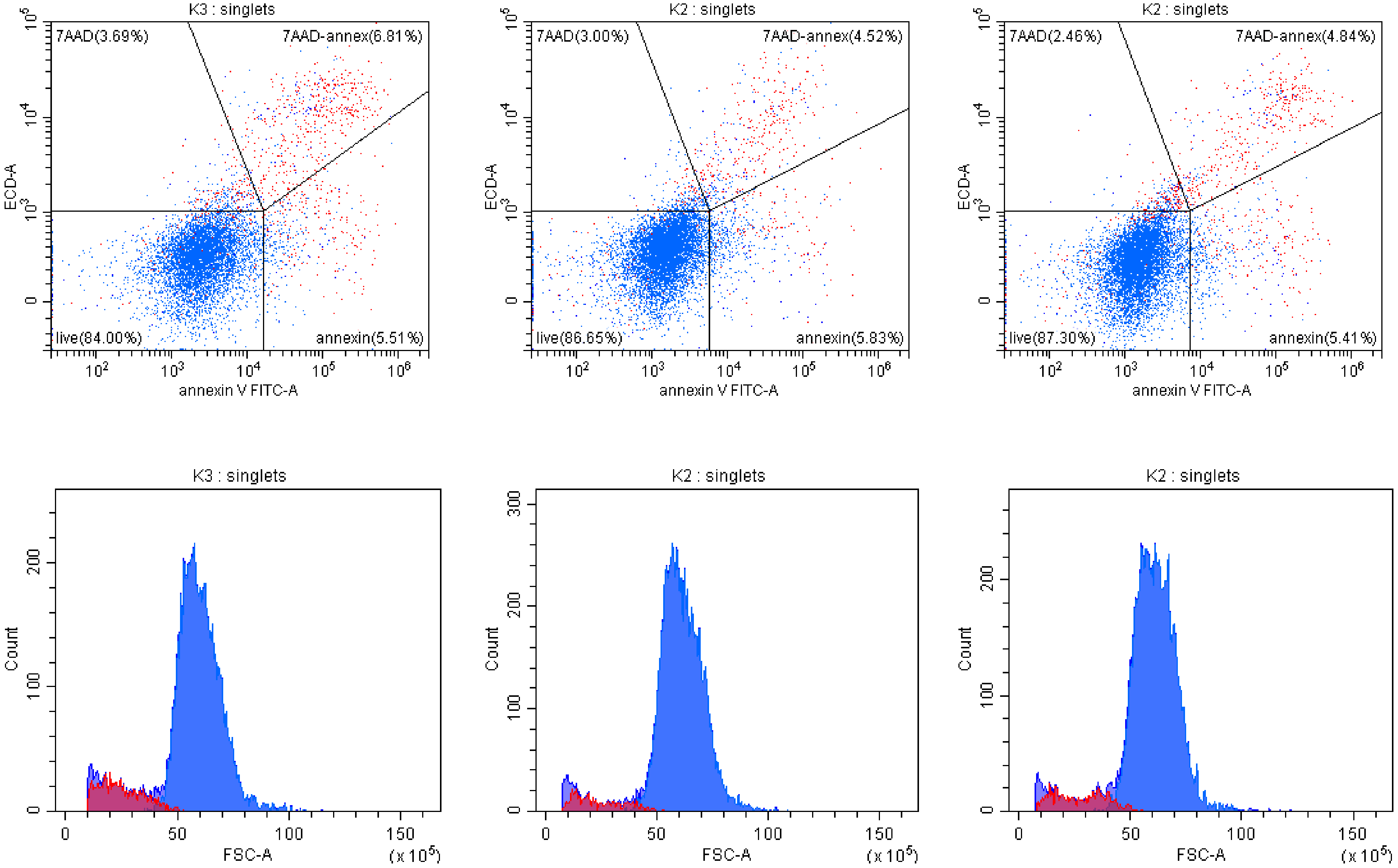

MDA-MB-231 PTX 2nM

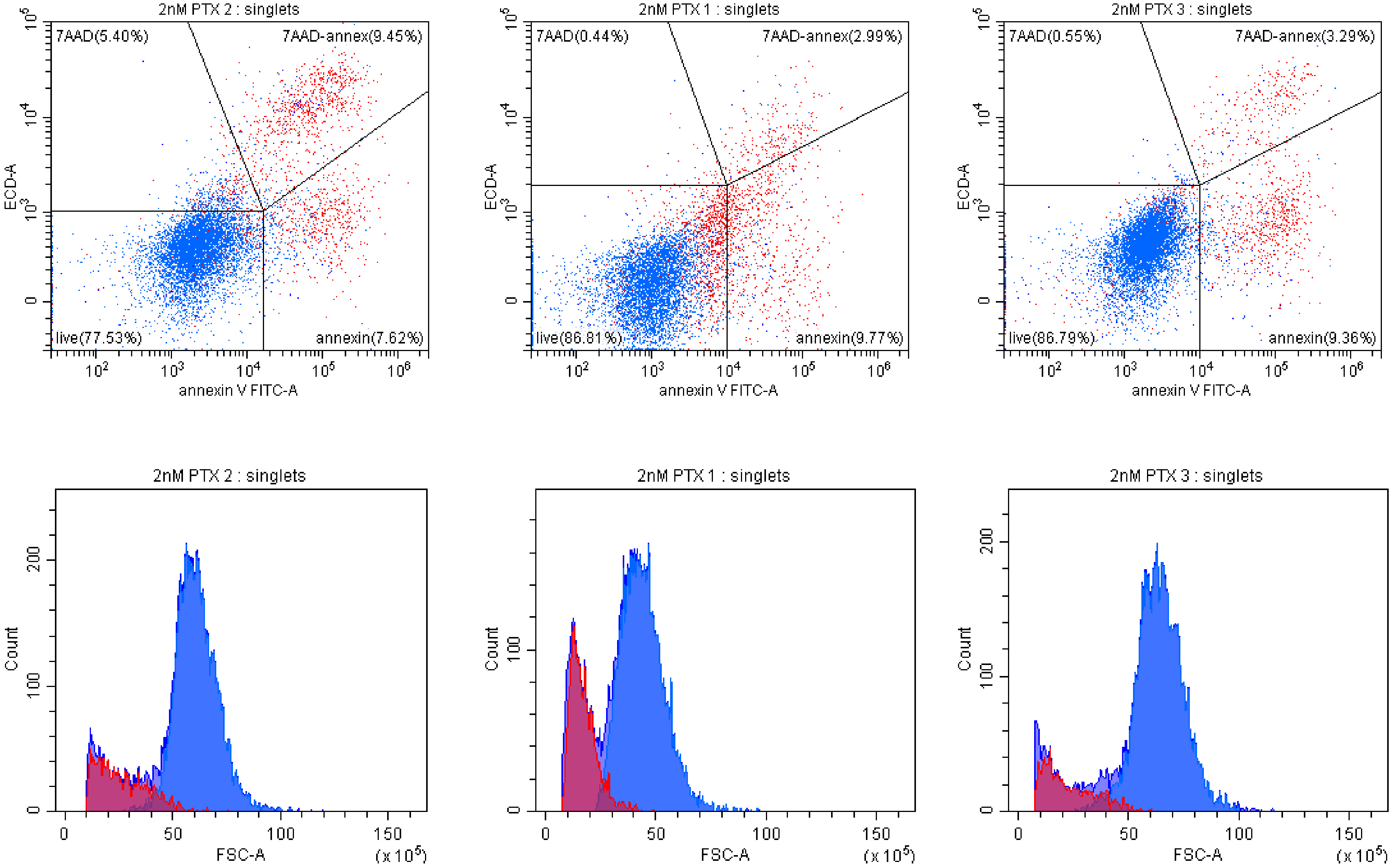

MDA-MB-231 PTX 20 nM

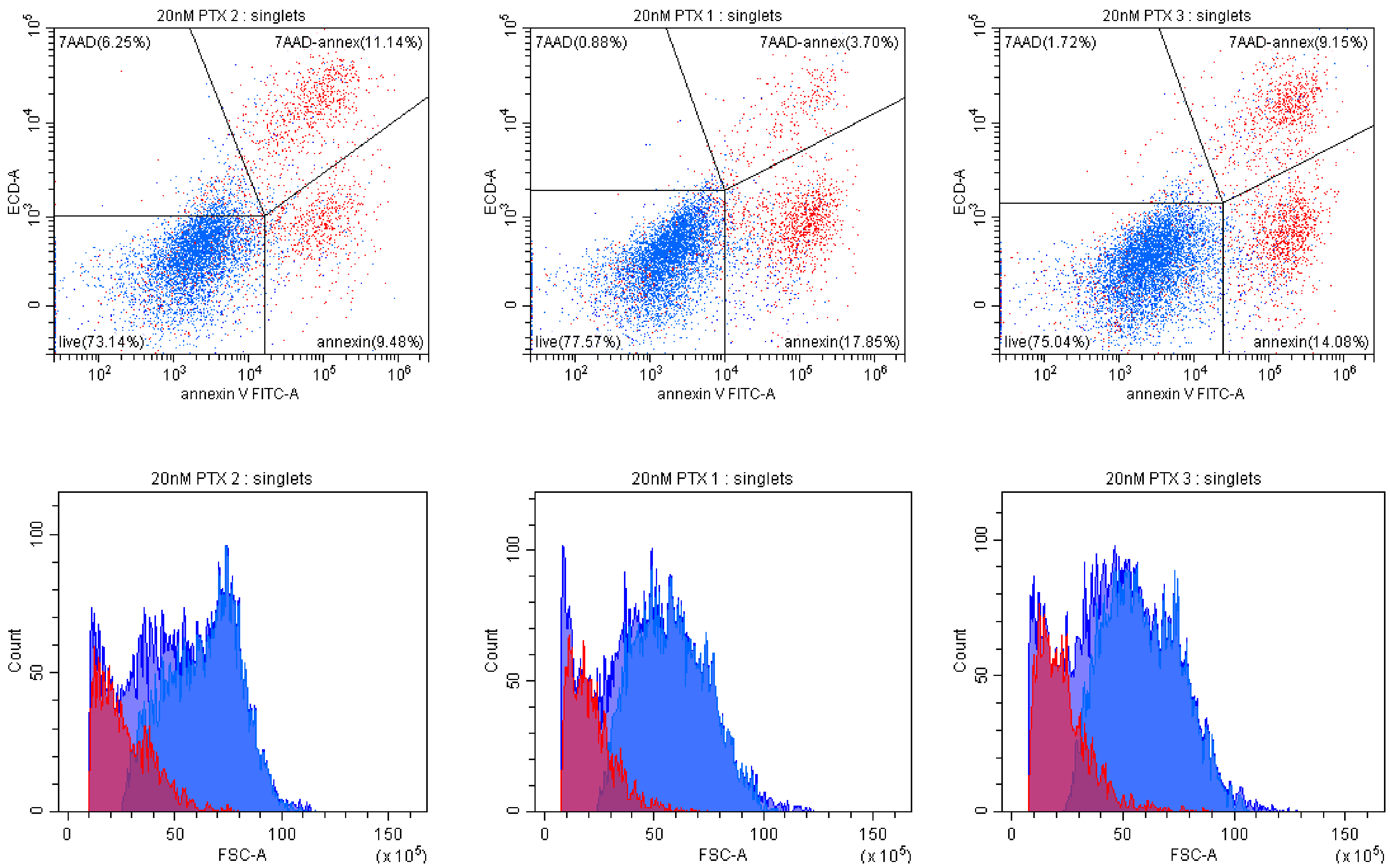

MDA-MB-231 PTX 200 nM

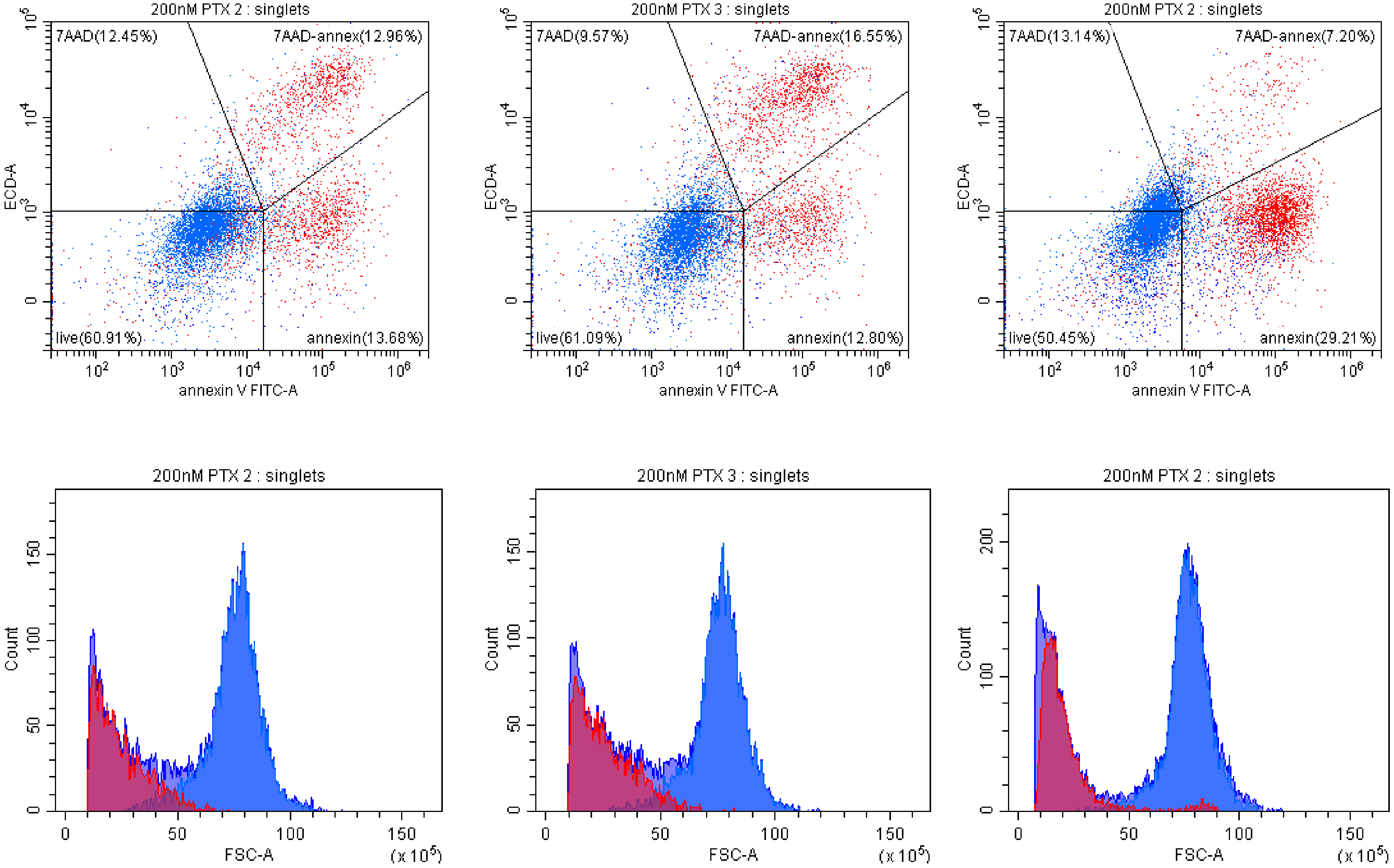

MDA-MB-231 GYY 100μM

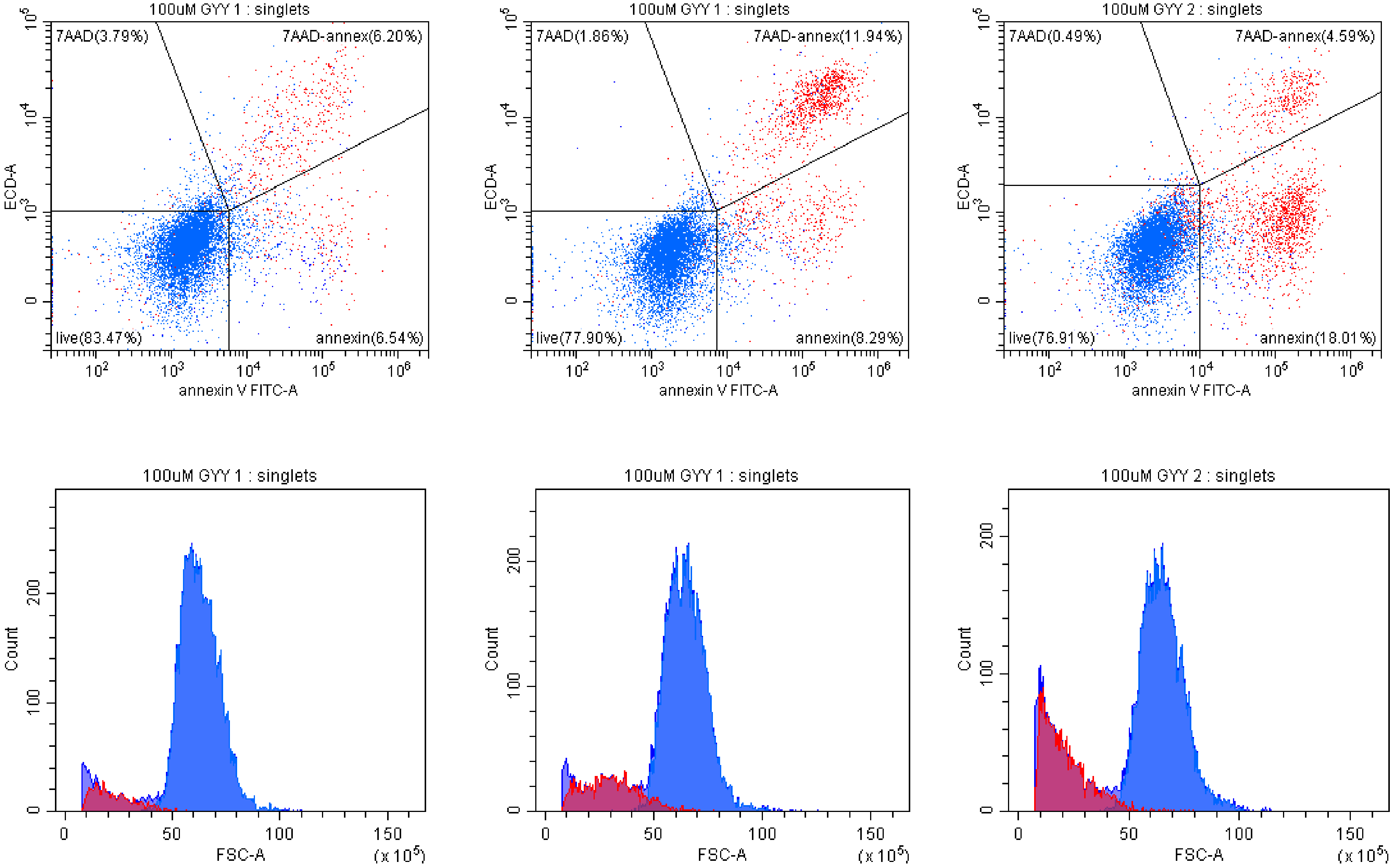

MDA-MB-231 PTX 2 nM+GYY 100μM

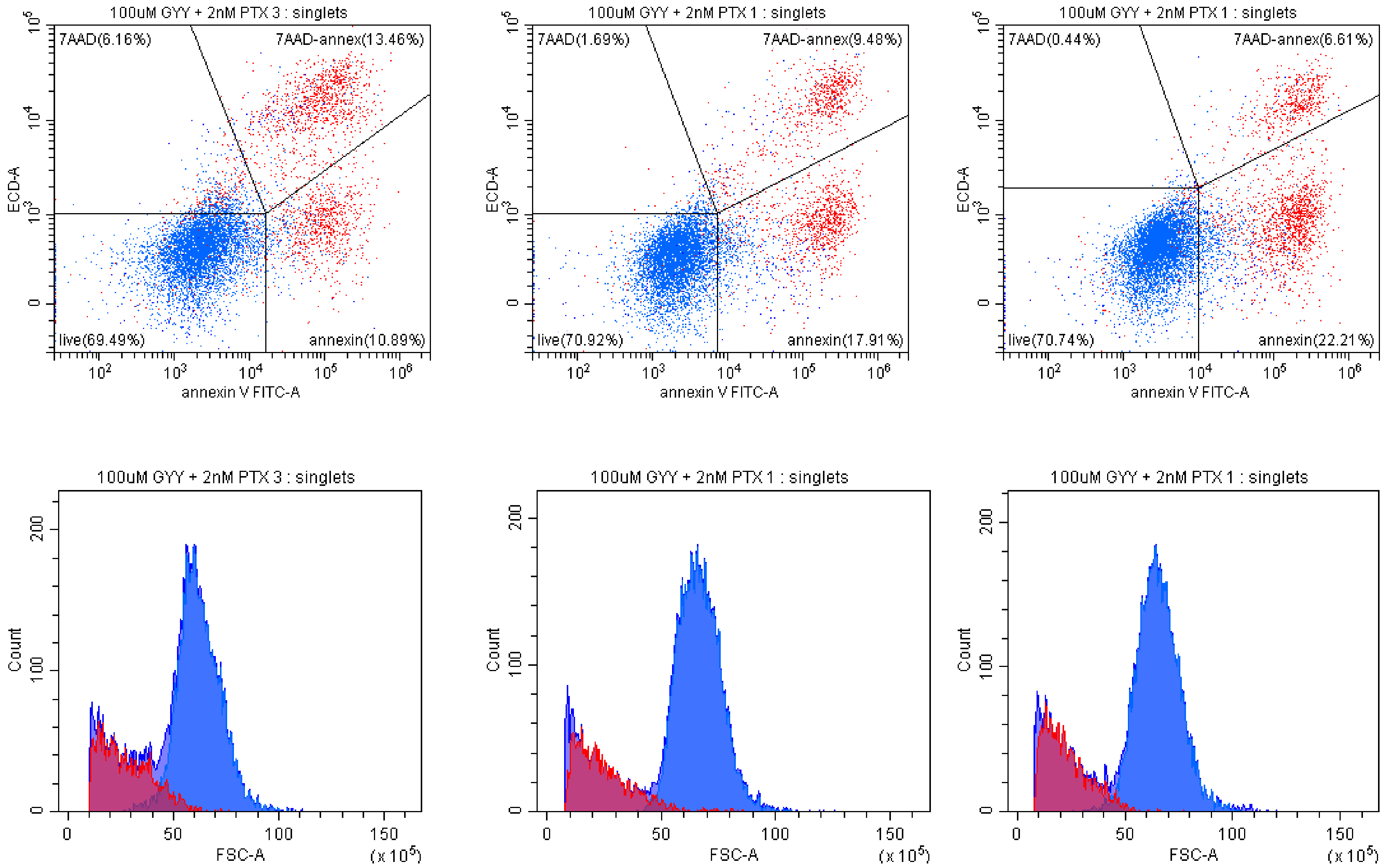

MDA-MB-231 PTX 20 nM+GYY 100µM

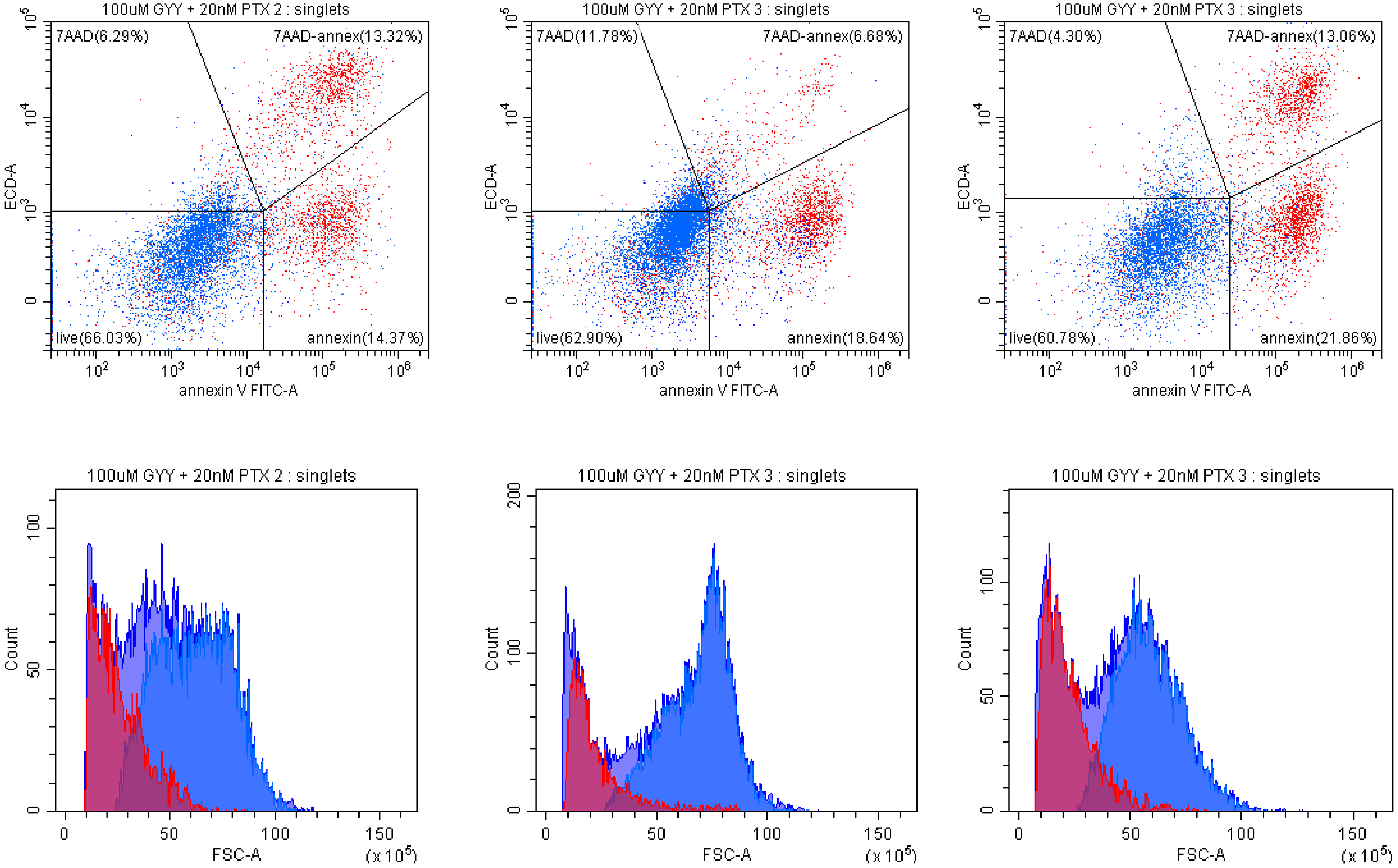

MDA-MB-231 PTX 200 nM+GYY 100μM

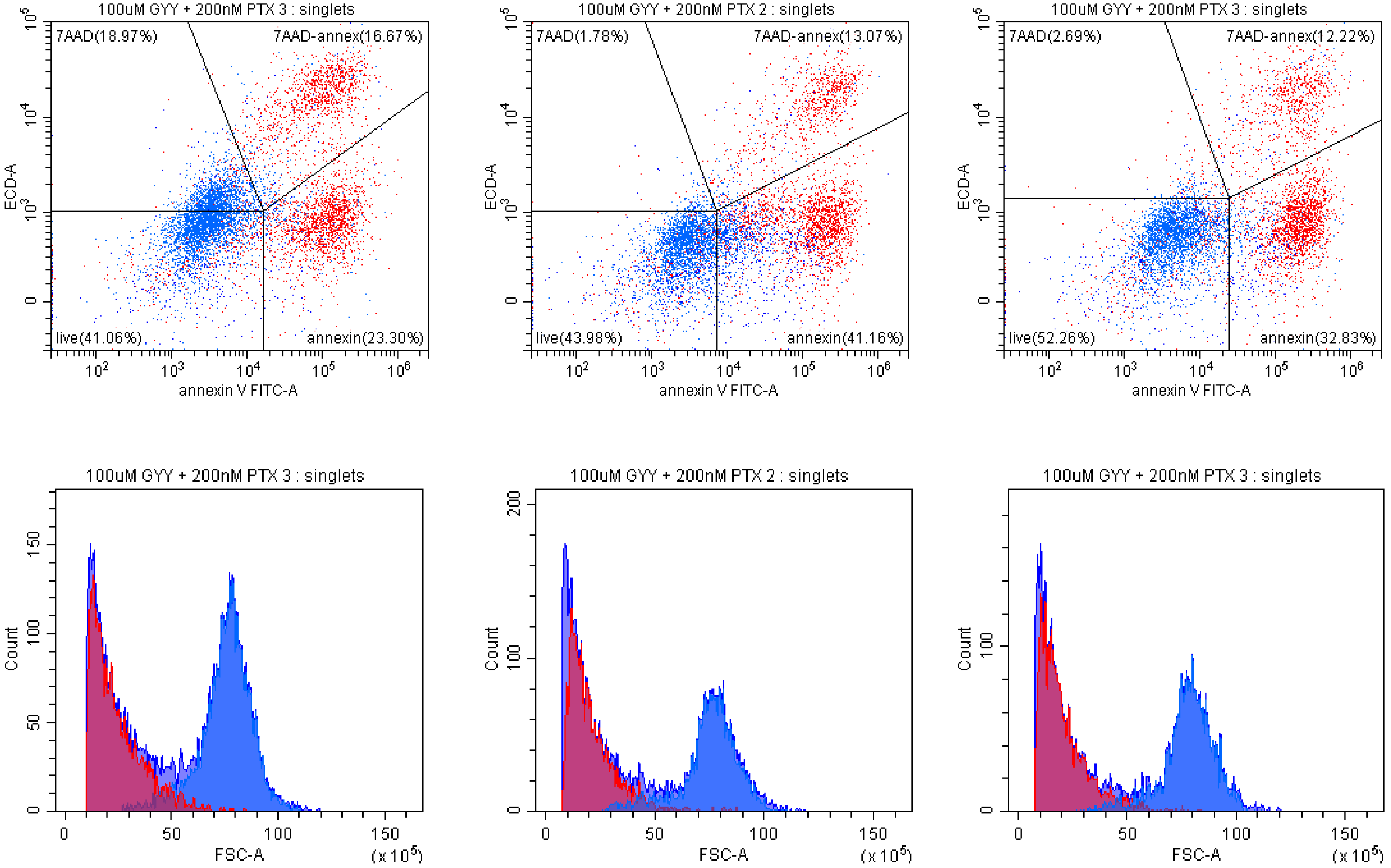

MDA-MB-231 DTT 1 mM

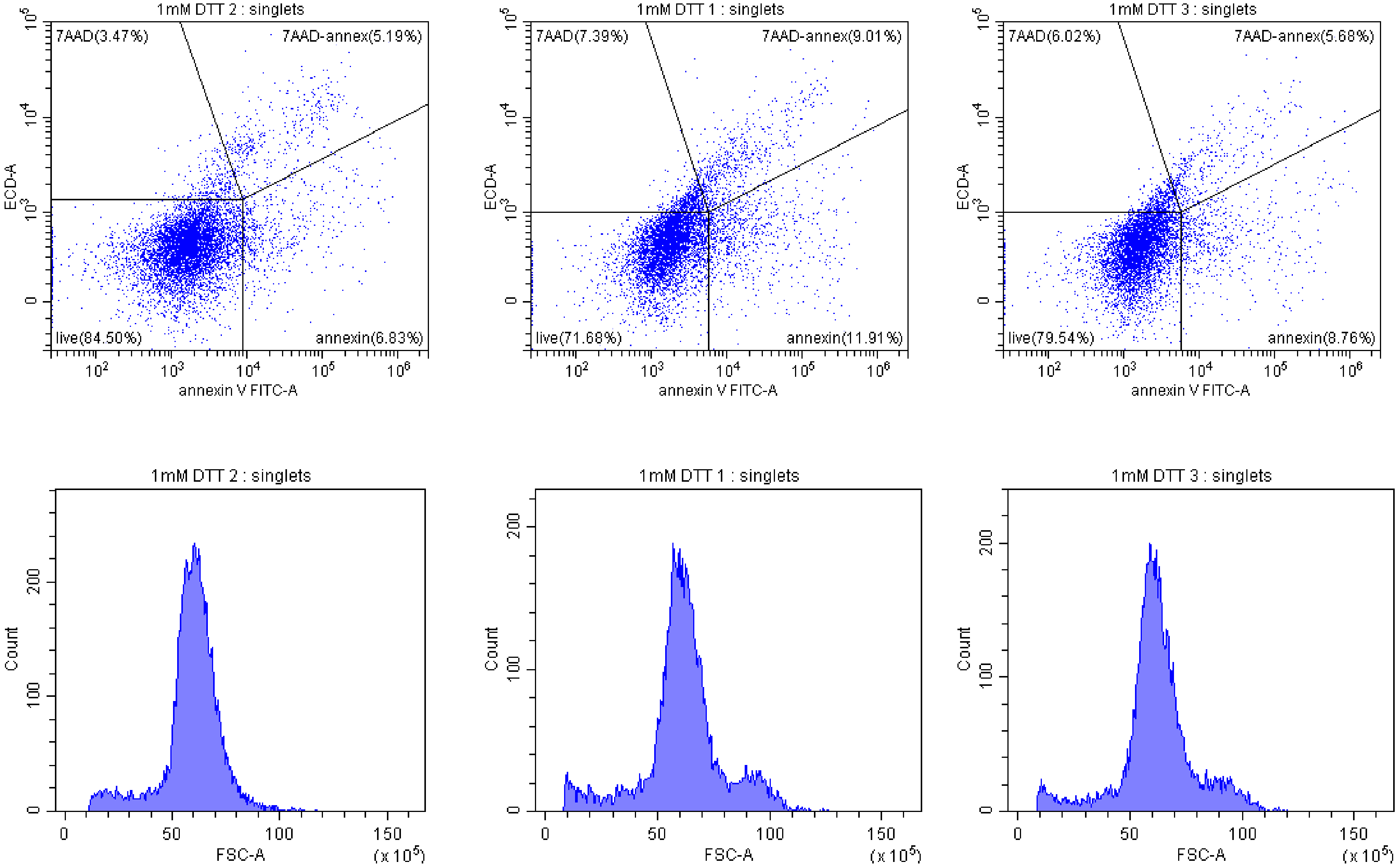

MDA-MB-231 PTX 20 nM+DTT 1 mM

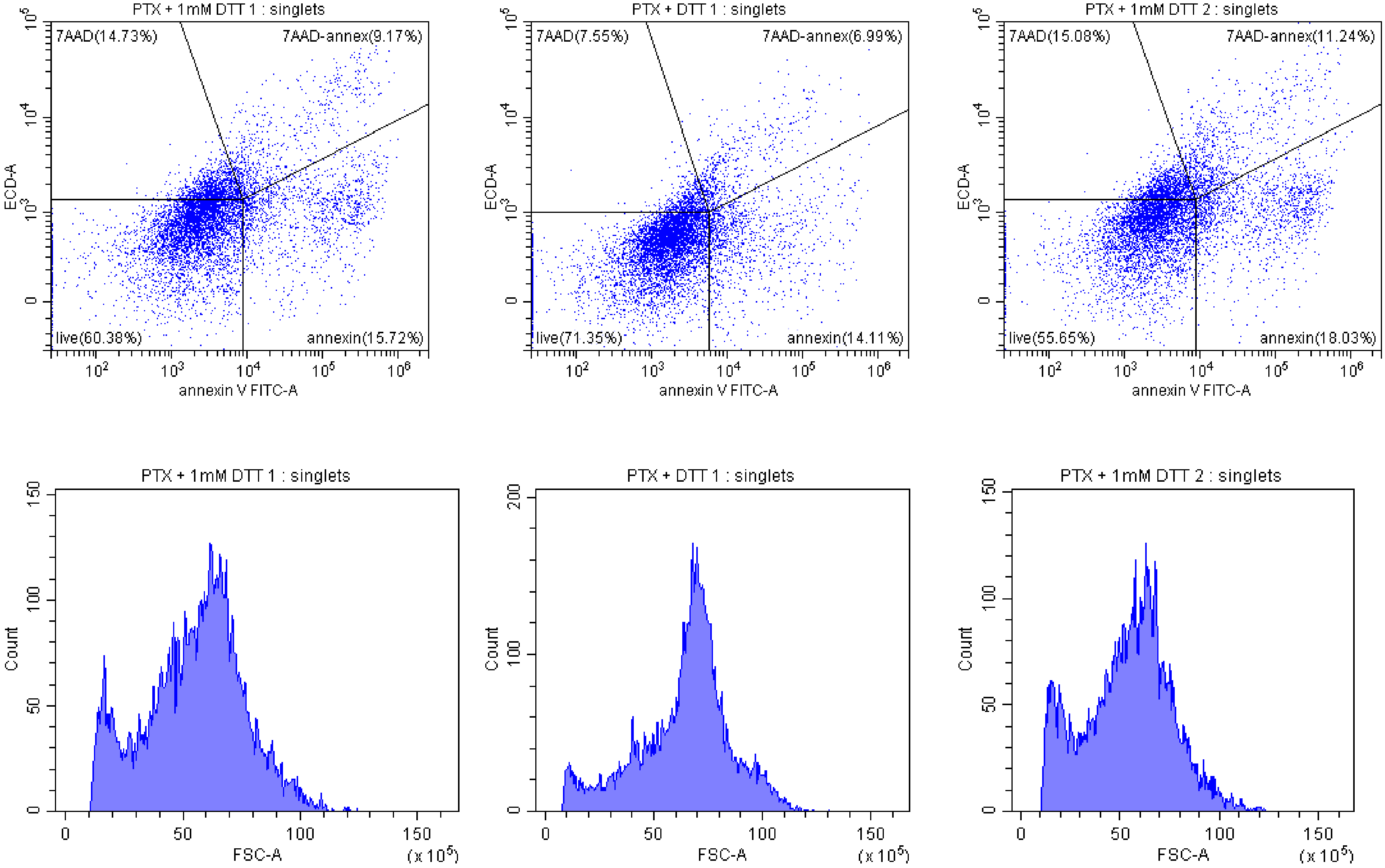

MDA-MB-231 TCEP 1 mM

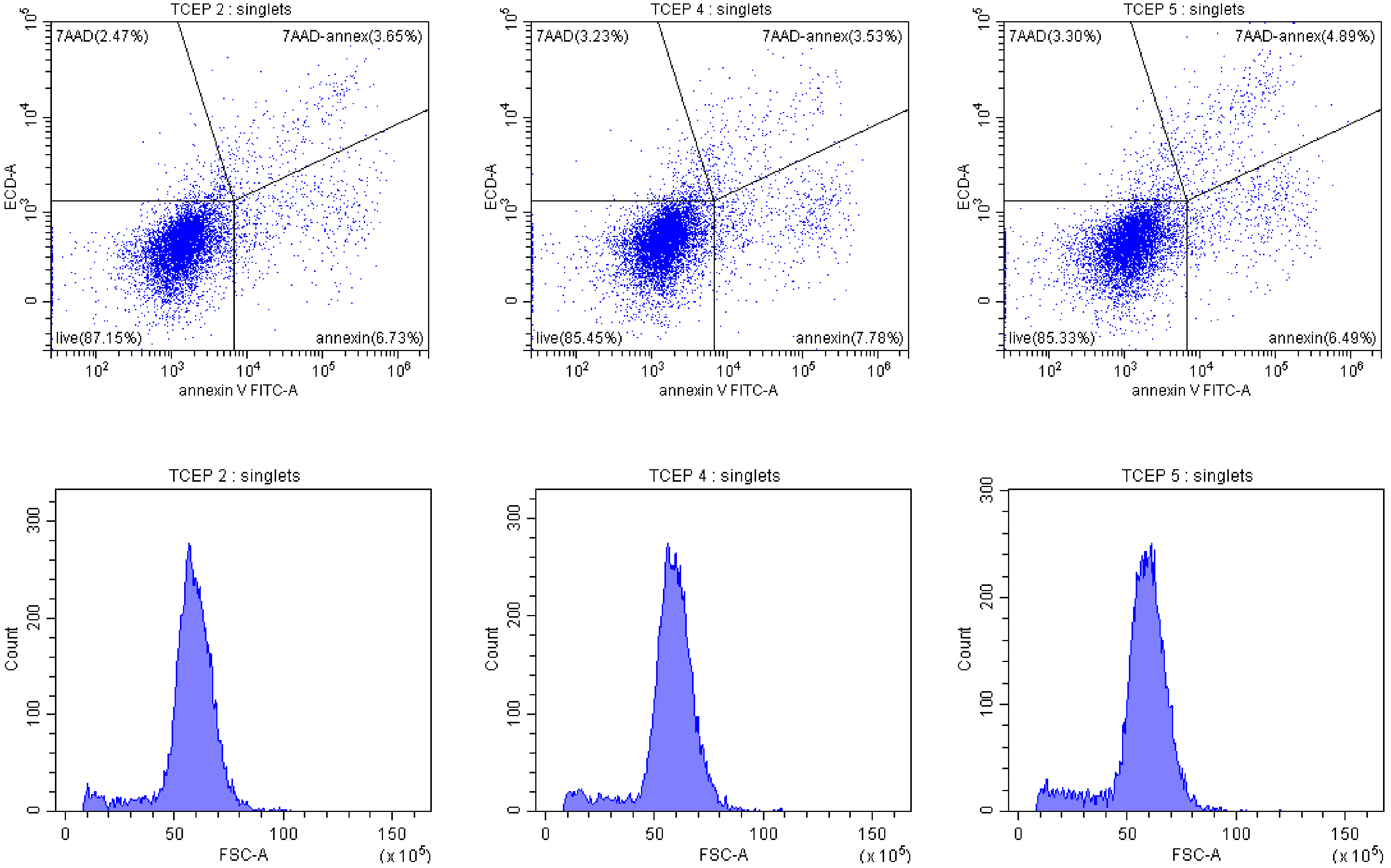

MDA-MB-231 PTX 20 nM+TCEP 1 mM

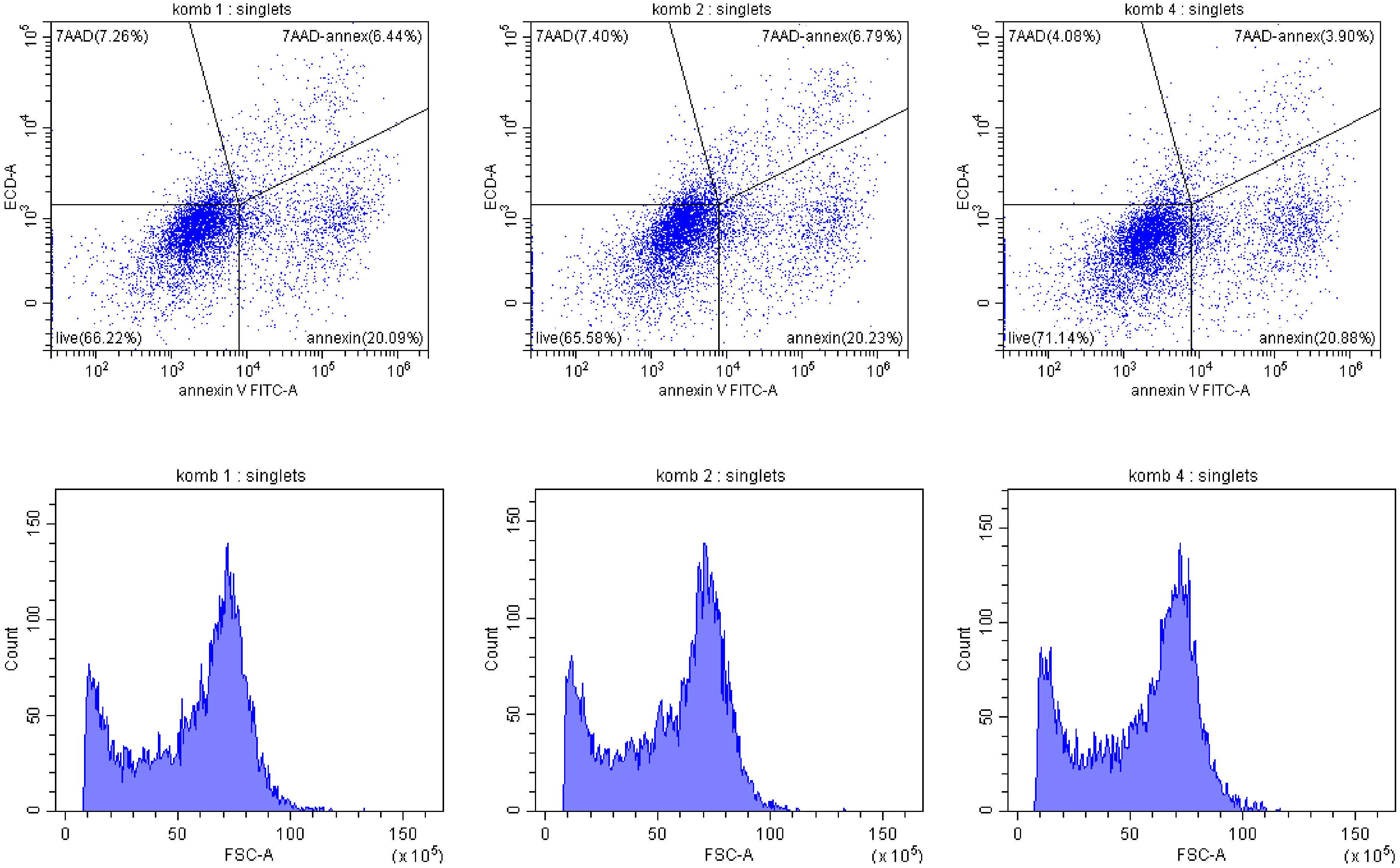

Figure S1D

MDA-MB-231

IP_3R1

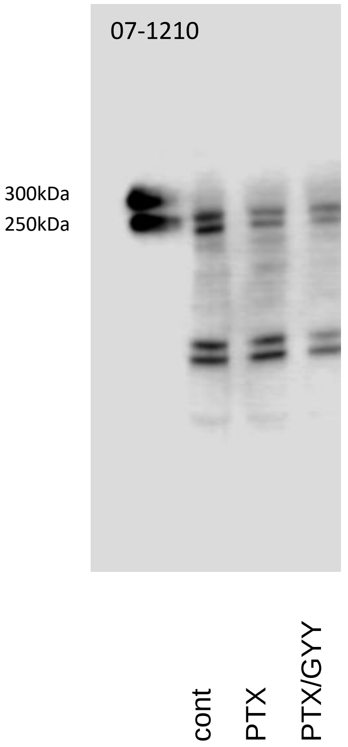

	IP3R1
cont	6390
PTX	5810
PTX/GYY	4550

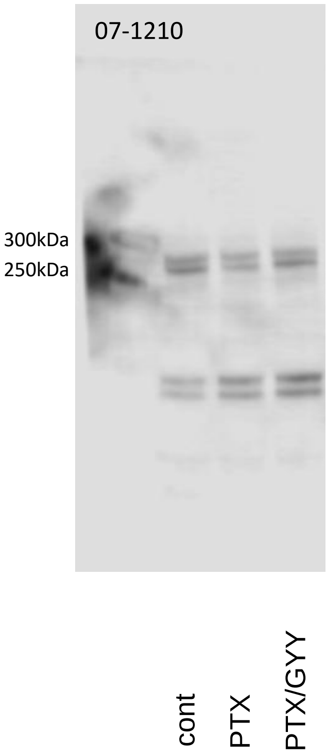

	IP3R1
cont	771
PTX	676
PTX/GYY	732

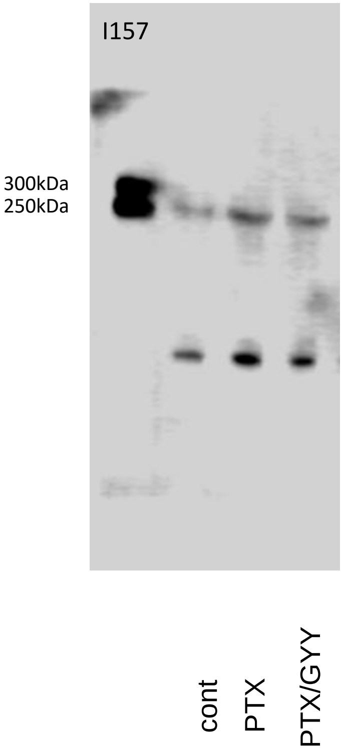

	IP3R1
cont	879
PTX	1560
PTX/GYY	1210

Figure S1D

MDA-MB-231

β-actin

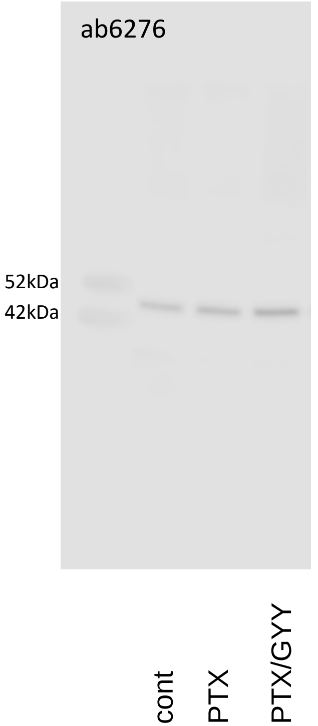

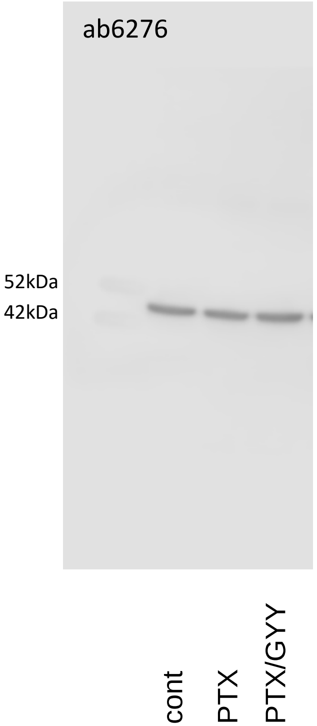

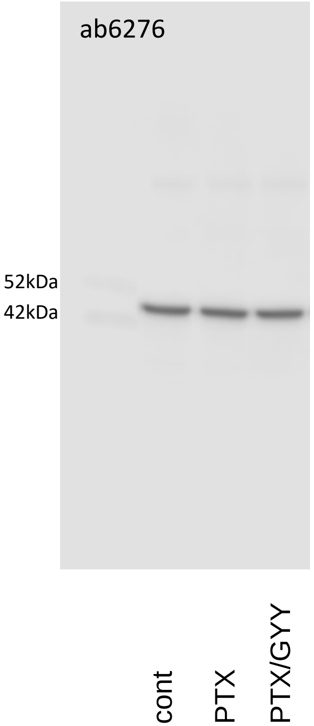

	**β-actin**
cont	1490
PTX	1950
PTX/GYY	2010

	**β-actin**
cont	7220
PTX	6940
PTX/GYY	7070

	**β-actin**
cont	32600
PTX	33100
PTX/GYY	32400

Figure S1D

JIMT1

IP_3R1

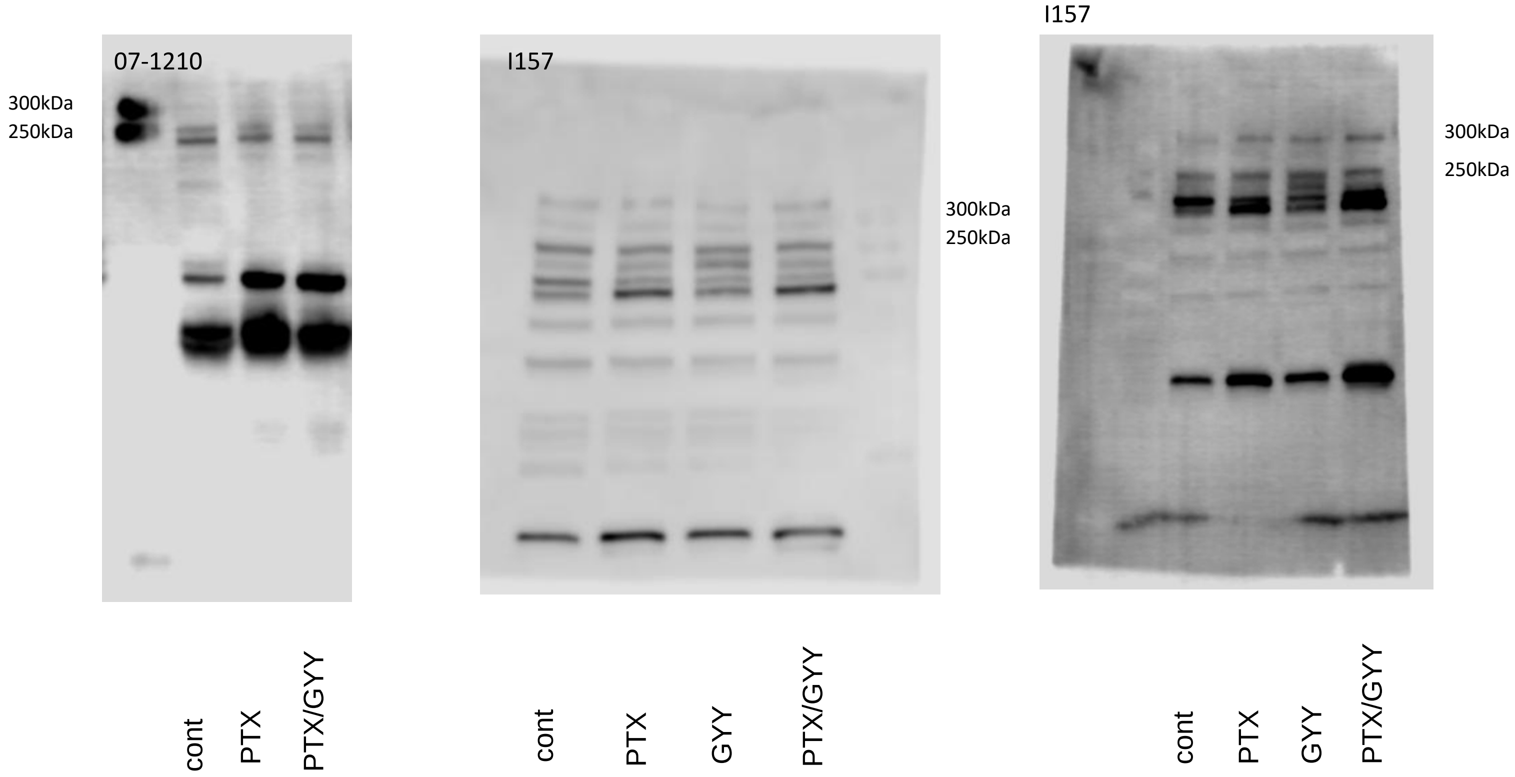

	IP3R1
cont	6210
PTX	5960
PTX/GYY	5620

	IP3R1
cont	690
PTX	640
GYY	670
PTX/GYY	664

	IP3R1
cont	2900
PTX	3400
GYY	3800
PTX/GYY	3500

Figure S1D

β-actin

JIMT1

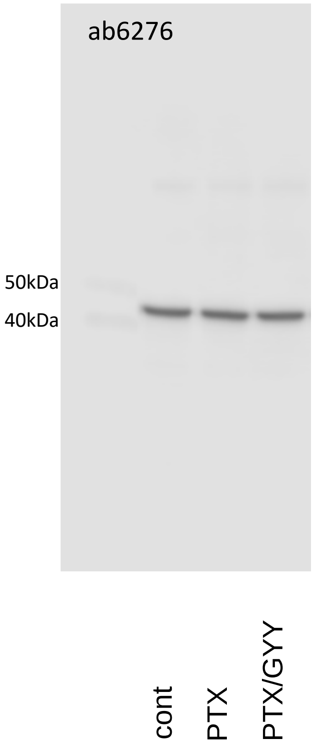

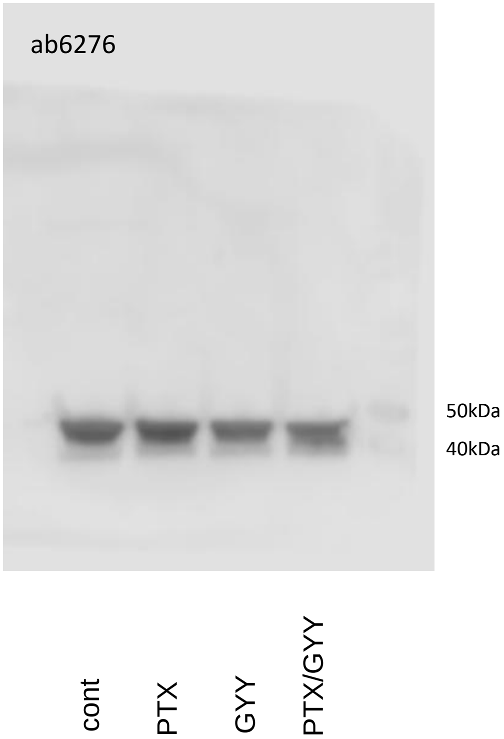

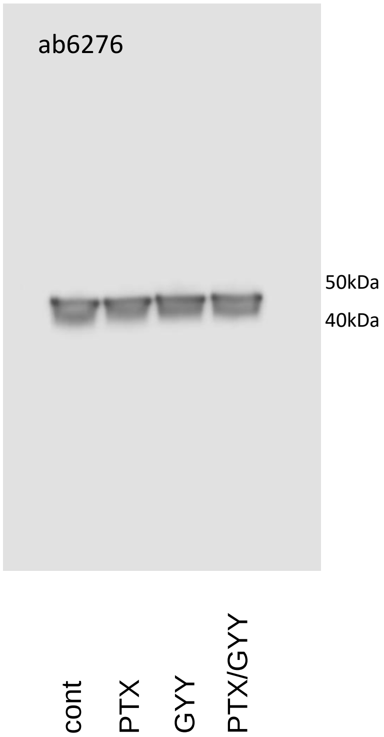

	**β-actin**
cont	326
PTX	331
PTX/GYY	324

	**β-actin**
cont	1400
PTX	1300
GYY	1300
PTX/GYY	1200

	**β-actin**
cont	7300
PTX	6900
GYY	7300
PTX/GYY	7100

FigureS2_pH_MDA-MB-231

							average	STDEV	S.E.M.
**MDA-MB-231_control**	6,84	6,80	6,67	6,65	7,02				
	7,09	6,49	6,58	6,58	6,67				
	7,01	6,22	6,51	6,58	6,78				
	7,14	6,65	6,40	6,56	6,89				
	7,11	6,68	6,46	6,75	6,81		6,72	0,23	0,05
**MDA-MB-231_PTX20**	6,90	6,47		6,55	6,42				
	6,95	6,37		6,61	6,54				
	6,98	6,44		6,55	6,60				
	7,00	6,37			6,49				
	6,28				6,49		6,59	0,23	0,06
**MDA-MB-231_GYY100**	6,75	6,49	6,35	6,15	6,75				
	6,97	6,53	6,31	6,25	6,75				
	6,94	6,35	6,41	6,37	6,82				
	6,81		6,32	6,43	6,73				
			6,11	6,38			6,52	0,26	0,06
**MDA-MB-231 PTX20 + GYY100**	6,13	6,04	5,80	6,08	6,26				
	6,53	6,09	5,92	6,09	6,52				
	6,72		6,13	6,10	6,44				
			6,27	6,22	6,57				
			6,22	6,13	6,04		6,21	0,23	0,05

FigureS2_pyruvate_MDA-MB-231

MDA-MB-231	control	PTX20	GYY100	PTX+GYY	
	1,88	1,20	1,15	0,79	
	1,85	1,28	1,44	0,68	
	1,53	1,09	1,26	0,89	
	2,12	1,10	1,28	0,78	
		1,25	1,14	0,85	
	1,85	1,18	1,25	0,80	average
	0,242	0,086	0,123	0,081	STDEV
	0,121	0,038	0,055	0,036	S.E.M.

FigureS2_lactate_MDA-MB-231

MDA-MB-231	control	PTX20	GYY100	PTX+GYY	
	3,06	4,84	7,84	12,10	
	3,21	4,89	7,37	9,62	
	1,75	5,81	9,42	8,88	
	1,59	6,16	6,75	13,64	
	1,91	6,57	8,51	11,73	
	2,75	6,06	5,25	14,04	
	2,38	5,72	7,52	11,67	average
	0,71	0,71	1,45	2,08	STDEV
	0,29	0,29	0,59	0,85	S.E.M.

FigureS2_pH_JIMT1

										average	STDEV	S.E.M.
**JIMT1_control**	6,67	7,33	6,91	7,01	6,66	6,83	6,91	6,76	6,56			
	6,78	7,42	6,80	7,04	6,55	6,78	6,79	6,72	6,38			
	6,58	7,32	6,58	6,94	6,50	6,69	6,57	6,65	6,21			
	6,49	7,00	6,60	6,91	6,37	6,66	6,53	6,57	6,19			
	6,43	7,02	6,49	6,85	6,39	6,54	6,30	6,56	6,06	6,69	0,29	0,04
**JIMT1_PTX20**	6,67	6,83	6,80	6,79	6,43	6,61	6,53	6,71	6,77			
	6,50	6,82	6,84	6,77	6,36	6,62	6,61	6,67	6,65			
	6,57	6,79	6,68	6,77	6,41	6,65	6,36	6,61	6,41			
	6,55	6,61	6,73	6,64	6,42	6,43	6,10	6,60	6,37			
	6,51	6,62	6,68	6,57	6,29	6,54	6,00	6,53	6,30	6,57	0,19	0,03
**JIMT1_GYY100**	6,47	7,10	6,95	6,94	6,58	6,69	6,02	6,81	6,77			
	6,70	7,25	6,77	6,88	6,52	6,83	6,70	6,66	6,56			
	6,53	7,04	6,75	6,73	6,43	6,71	6,45	6,53	6,28			
	6,46	6,86	6,55	6,66	6,35	6,61	6,21	6,50	6,00			
	6,29	6,88	6,64	6,59	6,30	6,52	6,02	6,43	5,98	6,59	0,29	0,04
**JIMT1 PTX20 + GYY100**	5,96	6,76	6,83	5,62	6,19	6,30	6,22	6,49	6,80			
	6,04	6,67	6,84	5,96	6,34	6,14	6,17	6,38	6,54			
	6,12	6,78	6,66	6,52	6,14	6,23	6,21	6,36	6,21			
	6,04	6,69	6,70	6,51	6,14	6,28	6,18	6,25	5,83			
	5,79	6,87	6,40	6,44	5,94	6,10	5,04	6,28	5,87	6,29	0,36	0,05

FigureS2_pyruvate_JIMT1

JIMT1	control	PTX20	GYY100	PTX+GYY	
	2,01	2,33	2,41	3,21	
	2,10	2,00	2,39	3,08	
	2,02	2,19	3,03	3,10	
	1,82	2,07	2,22	3,43	
	2,20	2,66	3,59		
	2,03	2,25	2,73	3,21	average
	0,141	0,264	0,572	0,160	STDEV
	0,063	0,118	0,256	0,080	S.E.M.

FigureS2_lactate_JIMT1

JIMT1	control	PTX20	GYY100	PTX+GYY	
	5,71	4,57	3,67	3,74	
	5,96	6,08	5,40	3,62	
	5,71	3,65	3,10	2,28	
	5,794	3,39	3,28	2,37	
	6,702	4,15	3,43	2,42	
	6,160	4,96	3,66	2,64	
	6,00	4,47	3,76	2,85	average
	0,38	0,98	0,84	0,66	STDEV
	0,16	0,40	0,34	0,27	S.E.M.

FigureS3_ROS_MDA-MB-231

MDA-MB-231	control	PTX20	GYY100	PTX+GYY	
	427	440	498	773	
	341	509	433	481	
	445	470	633	499	
	327	408	461	906	
	363	495	481	793	
	423	563	424	526	
	505	640	571	1300	
	421	658	453	1288	
	491	568	489	1127	
	459	491	592	813	
	552	544	529	570	
	493	853	707	428	
	554	677	521	616	
	571	637	425	636	
	456	630	771	791	
	428		675	396	
				440	
	453,50	572,20	541,44	728,41	average
	72,81	113,91	107,09	288,59	STDEV
	18,20	29,41	26,77	69,99	S.E.M.

FigureS3_ROS_JIMT1

JIMT1	control	PTX20	GYY100	PTX+GYY	
	580	932	497	592	
	634	859	509	683	
	695	670	551	600	
	606	649	469	609	
	637	721	552	572	
	567	850	447	624	
	471	740	569	841	
	492	542	544	681	
	515	551	497	777	
		630	494	923	
	577,44	714,40	512,90	690,20	average
	74,18	132,14	39,78	118,95	STDEV
	24,73	41,78	12,58	37,61	S.E.M.

Figure S3E

β-tubulin

MDA-MB-231

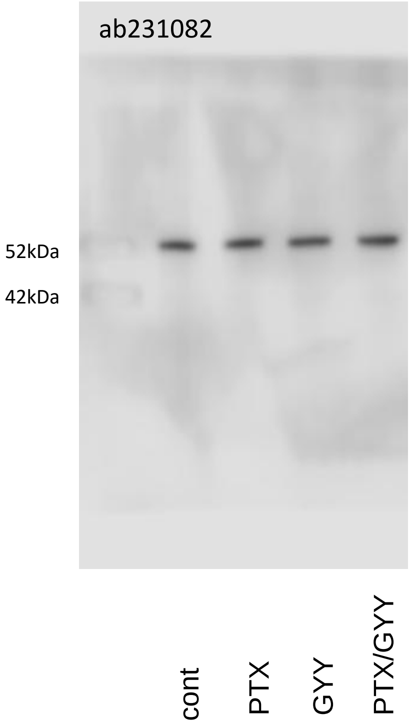

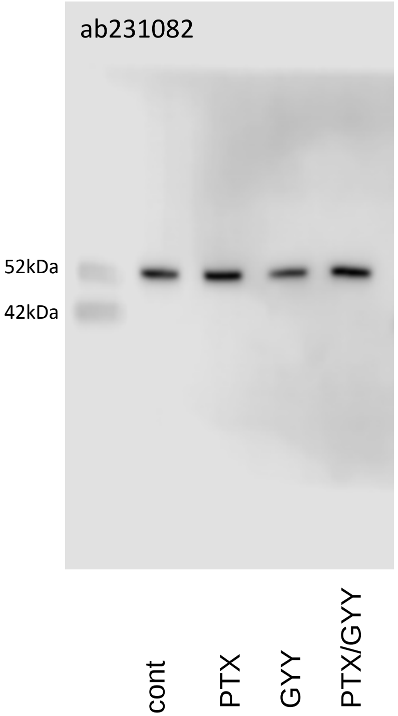

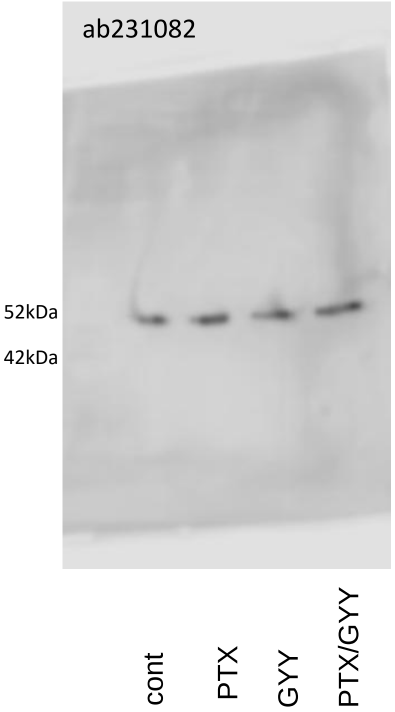

	**β-tubulin**
cont	3050
PTX	3710
GYY	4240
PTX/GYY	4350

	**β-tubulin**
cont	2080
PTX	2710
GYY	1990
PTX/GYY	2830

	**β-tubulin**
cont	2290
PTX	3140
GYY	4000
PTX/GYY	5160

Figure S3E

GAPDH

MDA-MB-231

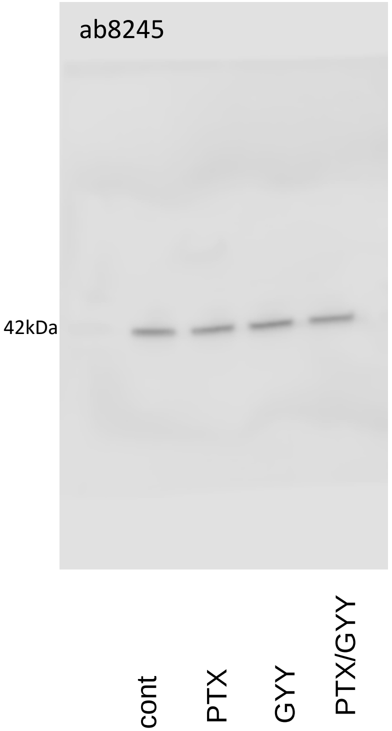

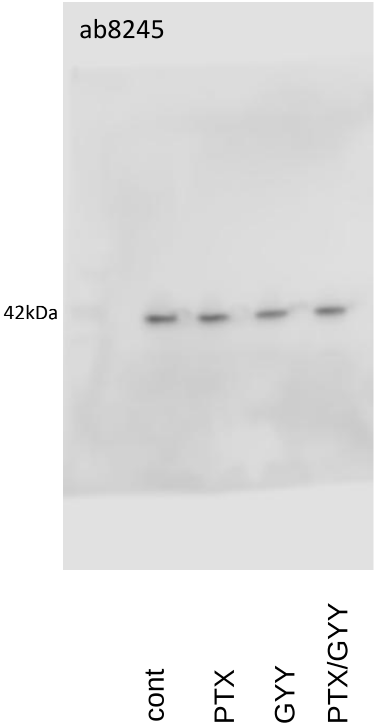

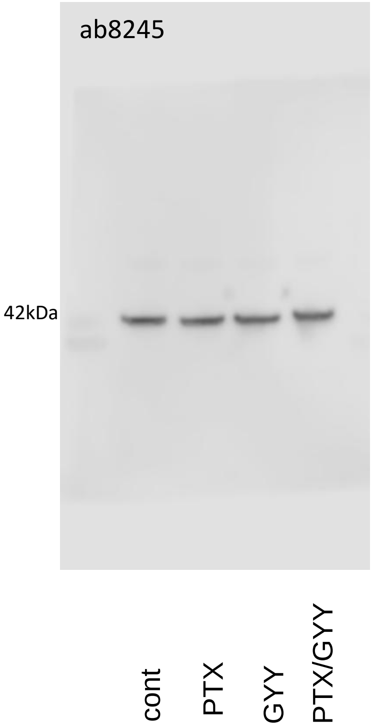

	**GAPDH**
cont	9290
PTX	9470
GYY	10900
PTX/GYY	9970

	**GAPDH**
cont	8250
PTX	7390
GYY	6700
PTX/GYY	6650

	**GAPDH**
cont	8250
PTX	8240
GYY	8300
PTX/GYY	7490

Figure S3E

β-tubulin

JIMT1

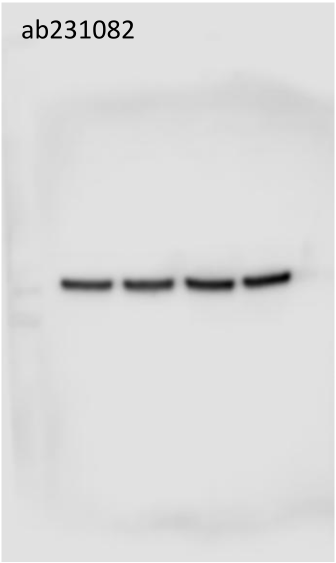

	β-tubulin
cont	78000
PTX	86000
GYY	98000
PTX/GYY	99000

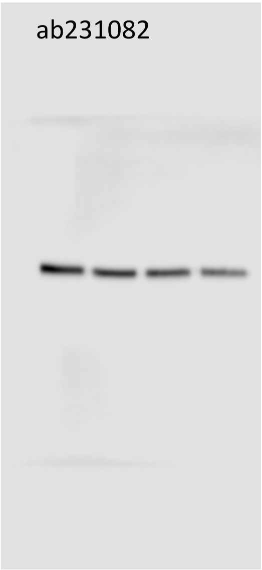

	β-tubulin
cont	32000
PTX	27000
GYY	24000
PTX/GYY	19000

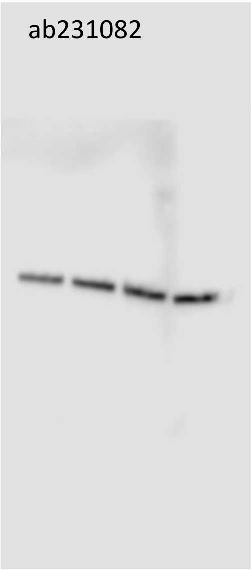

	β-tubulin
cont	21000
PTX	27000
GYY	32000
PTX/GYY	33000

Figure S3E

GAPDH

JIMT1

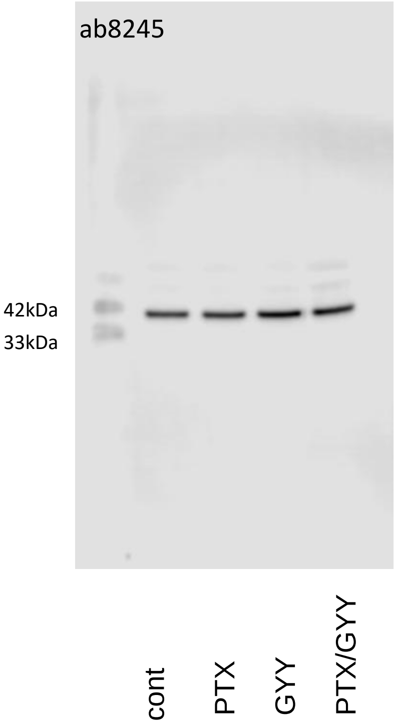

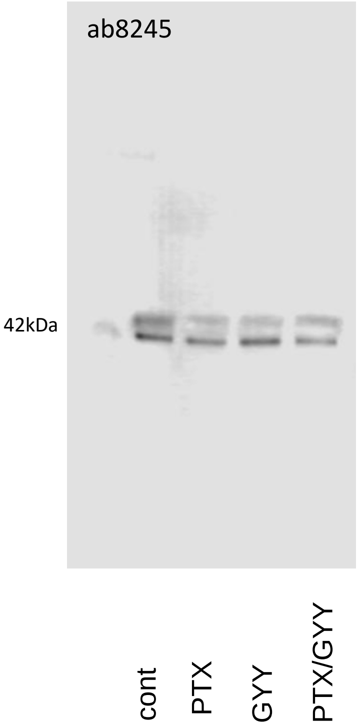

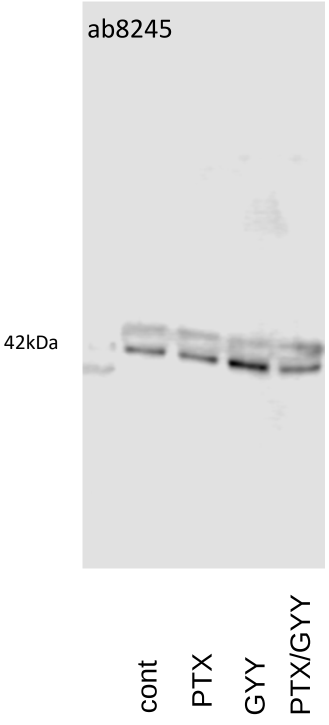

	**GAPDH**
cont	18000
PTX	19000
GYY	24000
PTX/GYY	20000

	**GAPDH**
cont	4000
PTX	3300
GYY	3700
PTX/GYY	2700

	**GAPDH**
cont	3500
PTX	3800
GYY	5900
PTX/GYY	4100

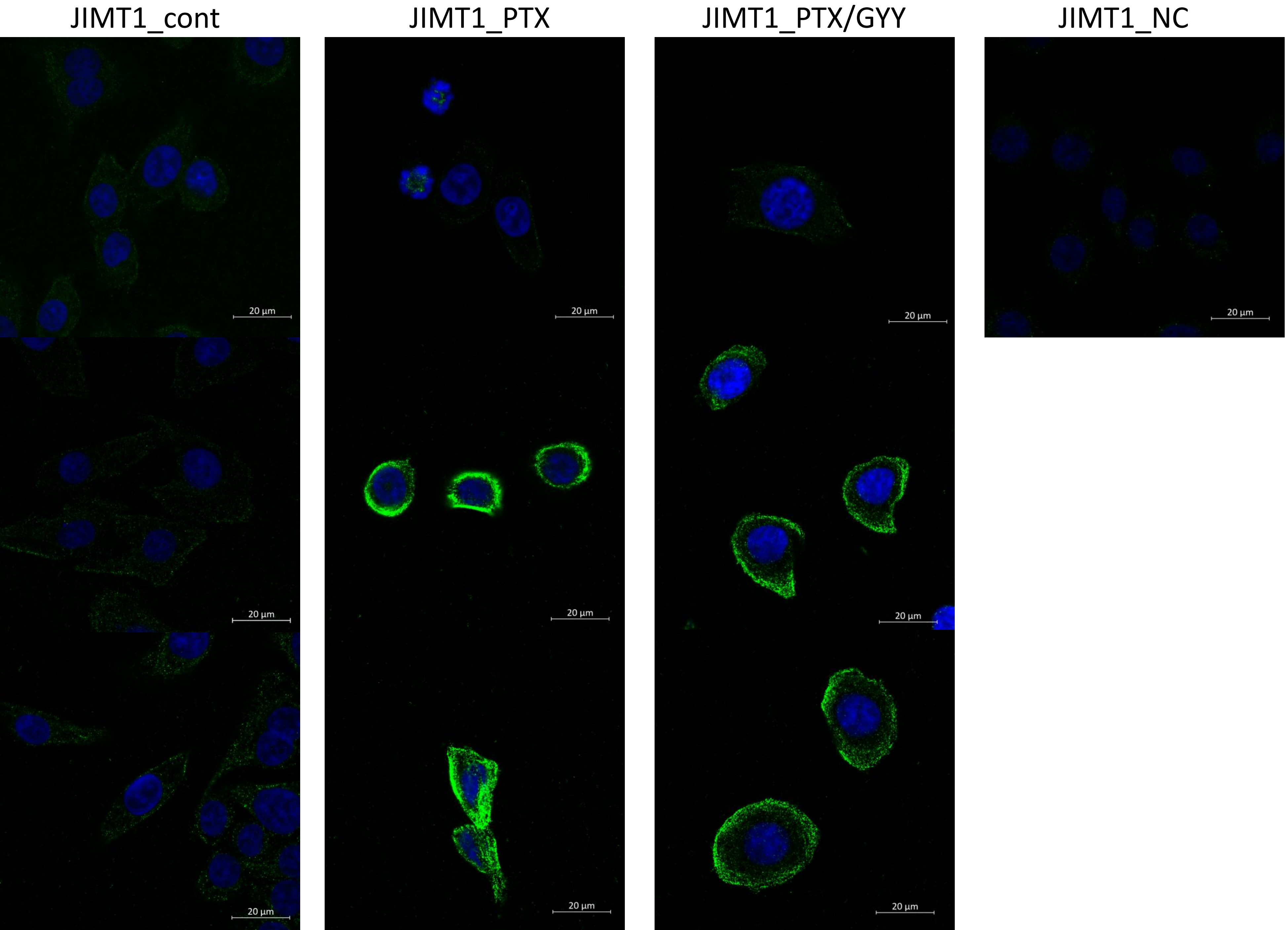

Figure 4_JIMT1

Green – beta tubulin
Blue – DAPI

FigureS5_scratch_MDA-MB-231

24 hours			
cont	PTX20	GYY100	PTX+GYY
25	8	35,7	2,9
25	8,3	14,3	5,9
17,4	8	28,5	5,9
21,7	12,5	21,4	5,9
25	9,5	20	5,5
16,6	4,8	8,7	5,5
25	9,5	13	11,1
25	9,5	21,7	5,9
25	4,3	25	14,2
28	7,1	33,3	21,4
31,25	13	30,7	14,2
33,3	17,4	16,6	14,2
33,3	13	16,6	4,3
33,3	16,6	28,6	4,3
17,8	21	26	20
11,5	15,7	22	20
53,8	26,6	17,6	12,5
50	28,5	22,2	8,7
		42,3	
		44	
		44	
		37	

48 hours			
cont	PTX20	GYY100	PTX+GYY
80	31	86,9	41,7
80	34,5	95,8	50
76,7	35,7	91,3	44,4
83	44,4	93,5	51
78	47,3	80,7	
78	55	92,3	
100	57,9	96	
100	52,6	100	

	cont	PTX20	GYY100	PTX+GYY
mean	27,66	12,96	25,87	10,13
SD	10,72	6,93	10,18	6,01
S.E.M	2,53	1,63	2,17	1,42

	cont	PTX20	GYY100	PTX+GYY
mean	84,46	44,80	92,06	46,78
SD	9,77	10,16	5,99	4,46
S.E.M	3,46	3,59	2,12	2,23

FigureS5_proliferation_MDA-MB-231

24 hours			
cont	PTX20	GYY100	PTX+GYY
0,896	0,68	0,773	0,639
0,909	0,685	0,795	0,496
0,946	0,652	0,723	0,68
0,823	0,652	0,679	0,67
0,737	0,743	0,678	0,462

48 hours			
cont	PTX20	GYY100	PTX+GYY
0,873	0,607	0,701	0,332
0,816	0,617	0,711	0,387
0,84	0,584	0,741	0,329
0,788	0,452	0,721	0,325
0,97	0,475	0,696	0,383

	cont	PTX20	GYY100	PTX+GYY
mean	0,862	0,682	0,730	0,589
SD	0,083	0,037	0,053	0,103
S.E.M	0,021	0,009	0,013	0,026

	cont	PTX20	GYY100	PTX+GYY
mean	0,857	0,547	0,714	0,351
SD	0,070	0,078	0,018	0,031
S.E.M	0,018	0,020	0,005	0,008

FigureS5_scratch_JIMT1

24 hours			
cont	PTX20	GYY100	PTX+GYY
13,64	13	8,3	4,3
14,2	9	12,5	4,3
10	4,5	12,5	4,3
13,6	4,5	8,7	4,5
22,7	18,2	16	15
27,3	13,6	16	5,2
23,8	17,3	16	10
27,3	13,6	20	15,7
10	10	15,3	20
21,7	14,2	28,5	20
13,6	10	15,4	20
25	14,2	13,3	14,3
25	14,2	13,3	12,5
23,8	9	35,7	8
41,17	13	7,4	13
29,4	9	7,4	13
23,5	16,7	30,4	
33,3	8,7	26	
30,4	16,6	34,6	
34,8	12,5	46,1	
26,9	6,2	40	
23,1	11,7	44	
17,6		23	
11,8		23	
22,2		16,7	
11,8		15,4	

48 hours			
cont	PTX20	GYY100	PTX+GYY
41	7,5	44,11	11,8
66,7	17,4	37,5	15,8
61,5	18,2	37,5	20
61,5	13	33,3	20
69,2	8,7	37,5	15
78,2	23,5	80,7	
87	29,4	76,9	
80,7	11,8	96	
69,2	12,5	100	
27,8		64,2	
29,4		61,5	
41,4		58,3	
55,5		53,8	

	cont	PTX20	GYY100	PTX+GYY
mean	22,22	11,80	20,98	11,51
SD	8,20	3,92	11,37	5,88
S.E.M	1,61	0,84	2,23	1,47

	cont	PTX20	GYY100	PTX+GYY
mean	59,16	15,78	60,10	16,52
SD	19,15	7,13	22,65	3,51
S.E.M	5,31	2,38	6,28	1,57

FigureS5_proliferation_JIMT1

24 hours			
cont	PTX20	GYY100	PTX+GYY
0,884	0,571	0,717	0,64
0,813	0,558	0,731	0,641
0,833	0,569	0,734	0,6
0,78	0,589	0,758	0,684
0,797	0,609	0,757	0,668

48 hours			
cont	PTX20	GYY100	PTX+GYY
0,778	0,358	0,753	0,281
0,714	0,376	0,777	0,266
0,72	0,346	0,697	0,23
0,698	0,322	0,61	0,246
0,915	0,396	0,8	0,301

	cont	PTX20	GYY100	PTX+GYY
mean	0,821	0,579	0,739	0,647
SD	0,040	0,020	0,018	0,032
S.E.M	0,010	0,005	0,004	0,008

	cont	PTX20	GYY100	PTX+GYY
mean	0,765	0,360	0,727	0,265
SD	0,089	0,028	0,076	0,028
S.E.M	0,022	0,007	0,019	0,007
